# Antibody responses to α-Gal in African children vary with age and site and are associated with malaria protection

**DOI:** 10.1038/s41598-018-28325-w

**Published:** 2018-07-03

**Authors:** Ruth Aguilar, Itziar Ubillos, Marta Vidal, Núria Balanza, Núria Crespo, Alfons Jiménez, Augusto Nhabomba, Chenjerai Jairoce, David Dosoo, Ben Gyan, Aintzane Ayestaran, Hèctor Sanz, Joseph J. Campo, Gloria P. Gómez-Pérez, Luis Izquierdo, Carlota Dobaño

**Affiliations:** 1 ISGlobal, Hospital Clínic-Universitat de Barcelona, Barcelona, Catalonia Spain; 2 CIBER Epidemiología y Salud Pública (CIBERESP), Barcelona, Spain; 3 Centro de Investigação em Saúde de Manhiça (CISM), Maputo, Mozambique; 4 Kintampo Health Research Center, Kintampo, Ghana

## Abstract

Naturally-acquired antibody responses to malaria parasites are not only directed to protein antigens but also to carbohydrates on the surface of *Plasmodium* protozoa. Immunoglobulin M responses to α-galactose (α-Gal) (Galα1-3Galβ1-4GlcNAc-R)-containing glycoconjugates have been associated with protection from *P. falciparum* infection and, as a result, these molecules are under consideration as vaccine targets; however there are limited field studies in endemic populations. We assessed a wide breadth of isotype and subclass antibody response to α-Gal in children from Mozambique (South East Africa) and Ghana (West Africa) by quantitative suspension array technology. We showed that anti-α-Gal IgM, IgG and IgG_1–4_ levels vary mainly depending on the age of the child, and also differ in magnitude in the two sites. At an individual level, the intensity of malaria exposure to *P. falciparum* and maternally-transferred antibodies affected the magnitude of α-Gal responses. There was evidence for a possible protective role of anti-α-Gal IgG3 and IgG4 antibodies. However, the most consistent findings were that the magnitude of IgM responses to α-Gal was associated with protection against clinical malaria over a one-year follow up period, especially in the first months of life, while IgG levels correlated with malaria risk.

## Introduction

Carbohydrates have not classically been considered to be significantly involved in adaptive immune responses, mostly being described as T cell-independent antigens that fail to induce immunological memory and immunoglobulin (Ig) class-switching^1^. However, studies of carbohydrate-based vaccines in mice have shown a dominant IgM response^2^ with some IgG production^3^. Since the early 1990s, naturally occurring glycoproteins^4^, glycolipids^5^, and even protein-free polysaccharides^6^ have been shown to be important components of the adaptive repertoire and, currently, polysaccharide-based conjugate vaccines are widely used to provide protective immunity against bacterial meningitis^7^. At present, there are no vaccines in use against complex human parasites, and there is a need to expand the pipeline of targets of protective immunity against malaria and other neglected diseases. The investigation of parasite glycosylation may provide new opportunities for the discovery of novel vaccine candidates against such diseases^8^.

The immune response against the malaria parasite *Plasmodium falciparum* has been mainly assessed against protein antigens. However, besides glycosylphosphatidylinositol (GPI) anchors^9^, the immunogenicity of carbohydrates allegedly located in the surface of this parasite is largely underappreciated since the parasite seems to have lost many of the genes required to elaborate complex carbohydrates^10^. Nevertheless, recent works showed the presence of precursors involved in glycoconjugate biosynthesis^11,12^ and identified new glycosylations in the parasite surface^13–15^. Some of these sugars modify important antigens in the fight against malaria, such as the circumsporozoite surface protein (CSP), which is the main component of the RTS,S vaccine^16^ and is O-fucosylated in malaria sporozoites. These post-translational modifications may alter protein antigenicity, being relevant for vaccine design.

It was recently demonstrated that antibodies against the classical α-Gal (Galα1-3Galβ1-4GlcNAc-R) epitopes confer protection against *Plasmodium* spp. infection, reducing malaria transmission by *Anopheles* mosquitoes^17^. This study showed that levels of IgG and IgM against α-Gal increased with age, and that IgM responses correlated with malaria incidence and were associated with protection against the disease^17^. On the contrary, IgG responses to α-Gal did not correlate with malaria exposure and were neither associated with protection^17^. Remarkably, other works have also reported the reduction of antigenicity of blood stage parasitic proteins after α-galactosidase treatment^18^. However, specific α-galactose containing glycans have never been isolated or structurally characterized in the malaria parasite.

Anti-α-Gal antibodies^19^ are produced against α-Gal epitopes, which are not expressed by humans due to the inactivation of the α-1,3-galactosyltransferase (α-1,3GT) in ancestral anthropoid primates^20^. Thus, anti-α-Gal antibodies are largely produced in response to cross-reactive epitopes present in commensal bacteria or food^21,22^, being the most abundant natural antibody in humans, constituting 1–5% of circulating IgM and IgG in healthy adults^21^. Anti α-Gal antibodies may also be produced in response to infection by pathogens expressing α-Gal, like the Gram-negative bacteria *Salmonella* spp. or the protozoan parasite *Trypanosoma* spp^22–25^. In fact, it has been argued that the selective pressure that removed this glycan from humans was exerted by an infectious agent like a virus, bacteria or protozoan expressing α-Gal epitopes, or an immunologically cross-reactive carbohydrate structure^21,26^.

In this study we investigated the anti-α-Gal response in children living in malaria-endemic areas of Mozambique (South East Africa) and Ghana (West Africa) who participated in clinical trials of the RTS,S/AS0 vaccine. We examined the effect of age, malaria transmission intensity (MTI) and other variables on anti-α-Gal IgM, IgG, IgG1, IgG2, IgG3 and IgG4 responses, and assessed their association with protection against clinical malaria and the factors affecting it.

## Results

### Pilot study of α-Gal IgG and IgM antibodies

IgM and IgG against α-Gal were first evaluated in children age 1–4 years from the RTS,S/AS02A phase 2b trial in Mozambique. Responses measured at the first visit (Month 0 [M0]) increased with age by 1.27 MFIs/year for IgM and by 1.48 MFIs/year for IgG (Table 1 and Supplementary Fig. 1). We observed a trend of IgG levels starting lower and increasing faster in Ilha Josina (cohort 2, high MTI) than in Manhiça (cohort 1, low MTI), reaching higher levels at age 4 years in Ilha Josina (Supplementary Fig. 1), however this trend was not statistically supported in the regression model, as site and age did not show a significant interaction (Table 1). These results suggest that IgM and IgG to α-Gal rise with age, and levels of IgM and IgG to α-Gal do not differ between neighborhoods of different MTI, or between malaria cases and controls in this small pilot study (data not shown).

**Table 1:** Regression models to assess the effect of age on anti-α-Gal antibody levels and interaction with site in children aged 1 to 4 years old from Manhiça and Ilha Josina.

	Coefficient	Std. Error	t value	Pr(>|t|)
	**IgM**			
(Intercept)	3.72402	0.15238	24.439	<2e-16
Age	0.10476	0.04851	2.16	**0.0358**
Site (Manhiça)	0.13553	0.21321	0.636	0.528
Age-site (Manhiça) interaction	−0.02768	0.06566	−0.422	0.6752
	**IgG**			
(Intercept)	3.48813	0.23936	14.573	<2e-16
Age	0.17339	0.07619	2.276	**0.0274**
Site (Manhiça)	0.2264	0.33492	0.676	0.5023
Age-site (Manhiça) interaction	−0.06506	0.10315	−0.631	0.5312

Data correspond to samples collected at baseline (M0) from children participants in the Mozambican RTS,S phase 2b clinical trial.

### Pattern of α-Gal antibody isotypes and subclasses in African children

**Figure 1:**
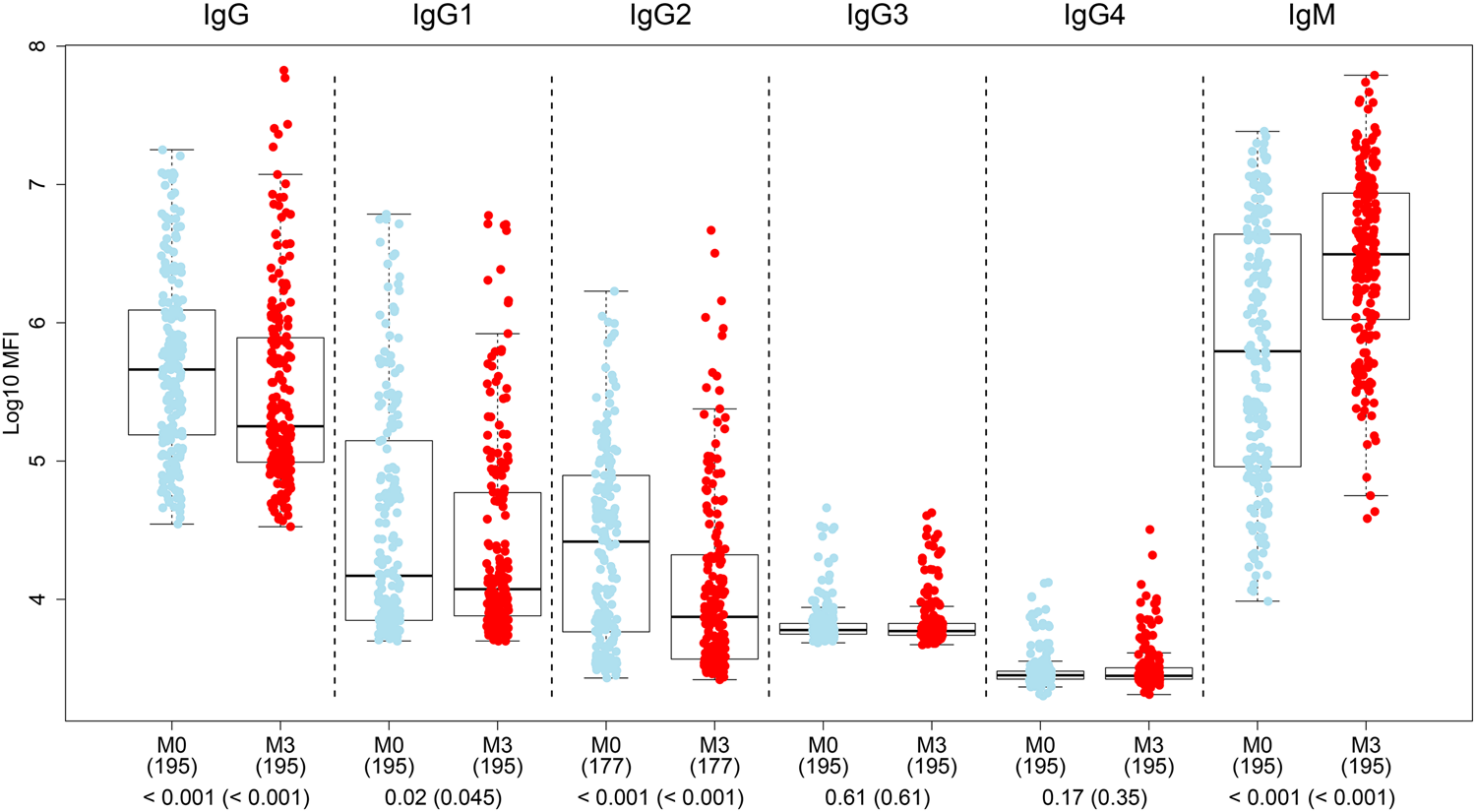
Antibody isotype and subclass responses to α-Gal by timepoint. Boxplots represent the median and interquartile ranges of IgM, IgG and IgG_1–4_ levels in infants and children from Manhiça and Kintampo (all together) participating in the RTS,S phase 3 trial, measured at the first recruitment visit (M0) and three months later (M3). Groups were compared through t-tests and p-values were adjusted for multiple comparisons through Benjamini-Hochberg and Holm (in parenthesis).

IgM, IgG and IgG_1–4_ against α-Gal were measured in children age ≤2 years from the RTS,S/AS01E phase 3 trial in Ghana and Mozambique, after confirming that vaccination did not have an effect on antibody response to α-Gal (Tables 2, 3 and S1). Thus, from here onwards, analyses were conducted regardless of vaccination group. IgM predominated over IgG responses. Among IgG subclasses, IgG1 and IgG2 tended to be higher than IgG3, and IgG4 was the lowest (Fig. 1). IgG, IgG1 and IgG2 were higher at the first study timepoint (M0) probably due to maternal transfer, and IgM was higher three months later (M3), reflecting continuous exposure to α-Gal.

**Table 2:** Factors affecting the anti-α-Gal response at month 3.

Antibody	Age*	Age cohort	Sex	Site	WAZ	HAZ	Hb	Exposure index	Maternal index	Prior episode^†^	Season^ǂ^	Vaccine	IgG at M0	IgM at M0
	Coef(CI) p-val	Coef(CI) p-val	Coef(CI) p-val	Coef(CI) p-val	Coef(CI) p-val	Coef(CI) p-val	Coef(CI) p-val	Coef(CI) p-val	Coef(CI) p-val	Coef(CI) p-val	Coef(CI) p-val	Coef(CI) p-val	Coef(CI) p-val	Coef(CI) p-val
**IgG**	4.16 (3.31;5.02) **<0.001**	403.47 (237.49;651.09) **<0.001**	−13.71 (−45.39;36.34) 1	76.7 (11.52;180) **0.047**	−17.01 (−32.47;2) 0.38	−4.84 (−22.02;16.12) 1	−0.2 (−15.32;17.62) 1	6.24 (0.64;12.16) 0.14	−7.33 (−12.39;1.97) **0.03**	17.99 (−43.6;146.87) 1	−10.38 (−66.78;141.74) 1	3.12 (−36.44;67.29) 1	449.62 (328.11;605.61) **<0.001**	138.22 (93.47;193.33) **<0.001**
**IgG1**	2.64 (1.65;3.64) **<0.001**	189.97 (84.24;356.36) **<0.001**	2.49 (−36.25;64.75) 1	91.76 (19.18;208.53) **0.03**	−6.95 (−24.97;15.41) 1	−6.96 (−24.32;14.39) 1	−1.56 (−16.97;16.72) 1	2.07 (−3.56;8.03) 1	−9.13 (−15.43;−2.35) **0.03**	−20.31 (−62.92;71.25) 1	26.21 (−54.86;252.9) 1	−28.76 (−56.76;17.38) 1	189.1 (108.36;301.14) **<0.001**	75.7 (38.54;122.82) **<0.001**
**IgG2**	3.15 (2.2;4.11) **<0.001**	173.79 (75.41;327.35) **<0.001**	−17.39 (−48.12;31.52) 1	7.01 (−33.26;71.6) 0.78	−7.91 (−25.31;13.53) 1	−9.31 (−25.68;10.67) 1	−1.6 (−16.44;15.87) 1	1.85 (−3.48;7.47) 1	0.02 (−5.72;6.11) 0.99	18.53 (−44.13;151.47) 1	2.8 (−62.52;181.97) 1	−3.38 (−40.82;57.75) 1	332.76 (225.44;475.47) **<0.001**	70.85 (35.43;115.53) **<0.001**
**IgG3**	0.84 (0.6;1.08) **<0.001**	54.04 (38.41;71.44) **<0.001**	−2.26 (−13.52;10.48) 1	31.71 (16.96;48.32) **<0.001**	0.22 (−5.2;5.96) 1	2.72 (−2.61;8.33) 1	−2.44 (−6.62;1.92) 1	−0.06 (−1.51;1.42) 1	−2.89 (−4.56;−1.19) **0.004**	−12.81 (−28.38;6.13) 1	−10.96 (−31.68;16.05) 1	2.39 (−10.03;16.54) 1	15.29 (5.29;26.25) **0.007**	21.22 (14.32;28.54) **<0.001**
**IgG4**	0.77 (0.56;0.98) **<0.001**	43.15 (29.89;57.77) **<0.001**	−3.69 (−13.62;7.39) 1	23.74 (11.18;37.72) **<0.001**	1.64 (−3.26;6.79) 1	2.51 (−2.23;7.49) 1	−2.71 (−6.42;1.15) 0.68	0.26 (−1.04;1.57) 1	−2.98 (−4.34;−1.6) **<0.001**	−11.52 (−25.72;5.39) 0.85	−14.58 (−32.47;8.07) 0.94	3.18 (−8.03;15.77) 1	10.14 (1.51;19.51) **0.03**	18.13 (12.09;24.49) **<0.001**
**IgM**	4.3 (3.56;5.05) **<0.001**	609.34 (412.38;882) **<0.001**	−36.58 (−58.33;−3.48) 0.2	102.99 (33.11;209.54) **0.006**	−21.63 (−35.18;−5.24) 0.07	4.85 (−12.83;26.11) 1	−16.45 (−28.11;−2.9) 0.12	10.6 (5.32;16.13) **<0.001**	−18.4 (−23.63;−12.82) **<0.001**	−23.5 (−61.42;51.67) 1	−57.25 (−82.84;6.54) 0.41	14.99 (−26.58;80.09) 1	49.35 (8.7;105.2) **0.03**	208.53 (163.58;261.14) **<0.001**

Multivariable linear models including phase 3 participants from both age groups and sites together. The coefficients indicate % change for a unit change in the predictor (95% confidence intervals [CI]). P-values are adjusted for multiple testing through Benjamini-Hochberg and Holm; those significant are in bold.

^*^Continous age in weeks. Age cohort (children vs infants). Sex (male vs female). Site (Manhiça vs Kintampo). WAZ (Weight-for-Age Z-score). HAZ (Height-for-Age Z-score). Hb (Baseline hemoglobin (g/dL). Exposure index (baseline anti-*P. falciparum* exposure IgM levels). Maternal index (baseline maternally transferred antibodies). ^†^Malaria episode between month 0 and month 3 (yes vs no). ^ǂ^Malaria transmission season at month 3 sample collection (low vs high). Vaccine (RTS,S vs comparator).

**Table 3:** Factors affecting the anti-α-Gal response at month 3 stratified by age group.

Antibody	Age*	Sex	Site	WAZ	HAZ	Hb	Exposure index	Maternal index	Prior episode^†^	Season^ǂ^	vaccine	IgG at M0	IgM at M0
	Coef(CI) p-val	Coef(CI) p-val	Coef(CI) p-val	Coef(CI) p-val	Coef(CI) p-val	Coef(CI) p-val	Coef(CI) p-val	Coef(CI) p-val	Coef(CI) p-val	Coef(CI) p-val	Coef(CI) p-val	Coef(CI) p-val	Coef(CI) p-val
**Infants**													
IgG	13.89 (−0.69;30.61) 0.31	−22.34 (−46.73;13.21) 0.93	44.7 (−1.77;113.16) 0.31	−5.4 (−21.29;13.7) 1	4.39 (−11.17;22.67) 1	−0.73 (−15.88;17.14) 0.93	−0.31 (−5.94;5.66) 1	−4.08 (−10.61;2.92) 0.73	0.12 (−48.12;93.23) 1	96.96 (6.32;264.89) 0.19	19.03 (−20.16;77.48) 0.95	235.96 (150.48;350.62) <**0.001**	−5.88 (−30.61;27.67) 1
IgG1	7.63 (−11.33;30.65) 1	−3.34 (−43.13;64.27) 1	56.1 (−9.3;168.67) 0.43	−3.6 (−25.45;24.65) 1	−4.63 (−23.92;19.55) 1	−18.33 (−34.97;2.57) 0.49	0.66 (−7.18;9.17) 1	−9.69 (−18.03;−0.49) 0.16	−51.53 (−80.44;20.07) 0.47	115.22 (−9.57;412.24) 0.41	−35.96 (−63.16;11.31) 0.68	−12.27 (−47.94;47.86) 1	−1.8 (−35.86;50.32) 1
IgG2	−7.24 (−20.69;8.47) 1	−4.21 (−36.69;44.93) 1	−26.77 (−51.99;11.71) 0.43	−6.47 (−23.15;13.83) 1	−4 (−19.18;14.03) 1	12.06 (−5.86;33.39) 0.72	−3.94 (−9.63;2.1) 1	0.62 (−6.87;8.71) 1	113.08 (1.23;348.53) 0.26	67.49 (−15.07;230.3) 0.54	33.11 (−13.8;105.54) 0.86	114.92 (45.65;217.14) <**0.001**	−27.37 (−47.97;1.38) 0.24
IgG3	0.52 |(−1.09;2.16) 1	3.44 (−0.98;8.06) 0.64	4.37 (−0.23;9.18) 0.31	0.03 (−2.09;2.19) 1	1.35 (−0.53;3.25) 0.95	−1.5 (−3.36;0.39) 0.58	−0.14 (−0.81;0.54) 1	−0.37 (−1.18;0.45) 0.75	−2.87 (−10;4.82) 0.90	9.43 (1.91;17.5) 0.08	2.43 (−2.21;7.29) 0.92	2.59 (−1.75;7.12) 0.73	−0.63 (−4.09;2.96) 1
IgG4	0.88 (−0.24;2.02) 0.49	−1.08 (−4.08;2.01) 1	2.6 (−0.59;5.9) 0.43	−1.19 (−2.64;0.28) 0.67	0.08 (−1.23;1.41) 1	−0.51 (−1.84;0.83) 0.9	0.02 (−0.45;0.49) 1	−0.77 (−1.32;−0.22) **0.03**	−5.25 (−10.08;−0.15) 0.26	1.07 (−3.97;6.37) 1	2.26 (−0.99;5.61) 0.86	0.23 (−2.77;3.33) 1	2.67 (0.21;5.19) 0.17
IgM	49.05 (25.69;76.76) <**0.001**	−50.77 (−69.89;−19.51) **0.03**	278.48 (138.23;501.3) <**0.001**	−16.91 (−34.95;6.13) 0.68	2.98 (−17.12;27.95) 1	−16.08 (−32.63;4.53) 0.58	5.17 (−2.67;13.64) 1	−16.68 (−23.74;−8.98) <**0.001**	−48.17 (−78.36;24.15) 0.47	7.17 (−53.99;149.65) 1	31.29 (−23.14;124.27) 0.95	49.53 (−8.99;145.69) 0.44	119.91 (50.3;221.78) <**0.001**
**Children**													
**Antibody**		Age*	**Sex**	**Site**	**WAZ**	**HAZ**	**Hb**	**Exposure index**	**Maternal index**	**Prior episode†**	**Vaccine**	**IgG at M0**	**IgM at M0**
		**Coef(CI) p-val**	**Coef(CI) p-val**	**Coef(CI) p-val**	**Coef(CI) p-val**	**Coef(CI) p-val**	**Coef(CI) p-val**	**Coef(CI) p-val**	**Coef(CI) p-val**	**Coef(CI) p-val**	**Coef(CI) p-val**	**Coef(CI) p-val**	**Coef(CI) p-val**
IgG		4.46 (2.15;6.82) **0.001**	21.57 (−44.06;164.22) 1	197.26 (41.24;525.65) **0.02**	−11.62 (−36.6;23.2) 1	−27.07 (−48.29;2.87) 0.43	31.26 (3.14;67.04) 0.14	−2.23 (−10.28;6.55) 1	−7.33 (−17.6;4.21) 0.78	2.96 (−67.76;228.75) 1	−14.86 (−62.48;93.21) 1	553.04 (407.38;740.51) **<0.001**	643.92 (247.07;1494.52) **<0.001**
IgG1		2.53 (0.1;5.02) 0.12	26.8 (−42.24;178.4) 1	196.13 (39.09;530.48) **0.02**	2.21 (−27.09;43.29) 1	−17.75 (−42.21;17.07) 1	33.39 (4.54;70.2) 0.13	−5.67 (−13.48;2.83) 0.73	−4.46 (−15.7;8.28) 0.93	5.06 (−67.62;240.84) 1	−18.89 (−64.64;86.05) 1	398.63 (255.6;599.19) **<0.001**	226.21 (39.11;664.94) **0.02**
IgG2		5.39 (2.83;8.02) **<0.001**	−12.98 (−64.11;110.98) 1	118.5 (−7.74;417.5) 0.15	2.75 (−29.53;49.81) 1	−23.1 (−47.82;13.33) 0.91	4.55 (−20.37;37.25) 1	−2.87 (−11.64;6.77) 1	−1.92 (−12.69;10.18) 0.93	−43.84 (−83.92;96.14) 1	−29.48 (−71.65;75.42) 1	491.83 (312.64;748.81) **<0.001**	481.29 (143.92;1285.23) **<0.001**
IgG3		0.01 (−0.74;0.76) 0.98	−3.81 (−24.22;22.09) 1	87.77 (54.22;128.61) **<0.001**	5.94 (−4.29;17.26) 1	1.14 (−9.17;12.63) 1	3.87 (−3.7;12.05) 0.96	−3.76 (−6.15;−1.32) **<0.001**	−3.76 (−9.35;2.16) 0.78	−27.28 (−48.73;3.14) 0.44	2.1 (−20.62;31.32) 1	19.74 (3.92;37.98) **0.03**	10.43 (−15.58;44.46) 0.93
IgG4		0.35 (−0.33;1.03) 0.63	−2.5 (−21.68;21.39) 1	64.9 (36.16;99.71) **<0.001**	9.34 (−0.26;19.88) 0.34	3.1 (−6.58;13.79) 1	1.4 (−5.45;8.74) 1	−2.65 (−4.91;−0.32) 0.13	−3.99 (−8.62;0.86) 0.51	−22.36 (−43.77;7.22) 0.61	4.16 (−17.34;31.23) 1	13.5 (−0.56;29.55) 0.06	7.65 (−15.91;37.82) 0.93
IgM		1.69 (0.61;2.79) **0.01**	12.46 (−21.66;61.45) 1	35.76 (−5.09;94.2) 0.15	−6.63 (−20.01;8.99) 1	−8.23 (−21.99;7.96) 1	13.11 (1.06;26.59) 0.14	−1.01 (−4.9;3.03) 1	−6.73 (−12.46;−0.62) 0.19	−22.59 (−54.82;32.63) 1	−3.92 (−34.45;40.83) 1	36.13 (10.01;68.47) **0.02**	183.54 (101.56;298.87) **<0.001**

Multivariable linear models including phase 3 participants from both sites stratifying by age group. The coefficients indicate % change for a unit change in the predictor (95% confidence intervals). P-values were adjusted for multiple comparisons through Benjamini-Hochberg and Holm; those significant are in bold.

*Continous age in weeks. Age cohort (children vs infants). Sex (male vs female). Site (Manhiça vs Kintampo). WAZ (Weight-for-Age Z-score). HAZ (Height-for-Age Z-score). Hb (Baseline hemoglobin (g/dL). Exposure index (baseline anti-*P. falciparum* exposure IgM levels). Maternal index (baseline maternally transferred antibodies). ^†^Malaria episode between month 0 and month 3 (yes vs no). ^ǂ^Malaria transmission season at month 3 sample collection (low vs high). Vaccine (RTS,S vs comparator).

### Effect of age on α-Gal antibody responses

**Figure 2:**
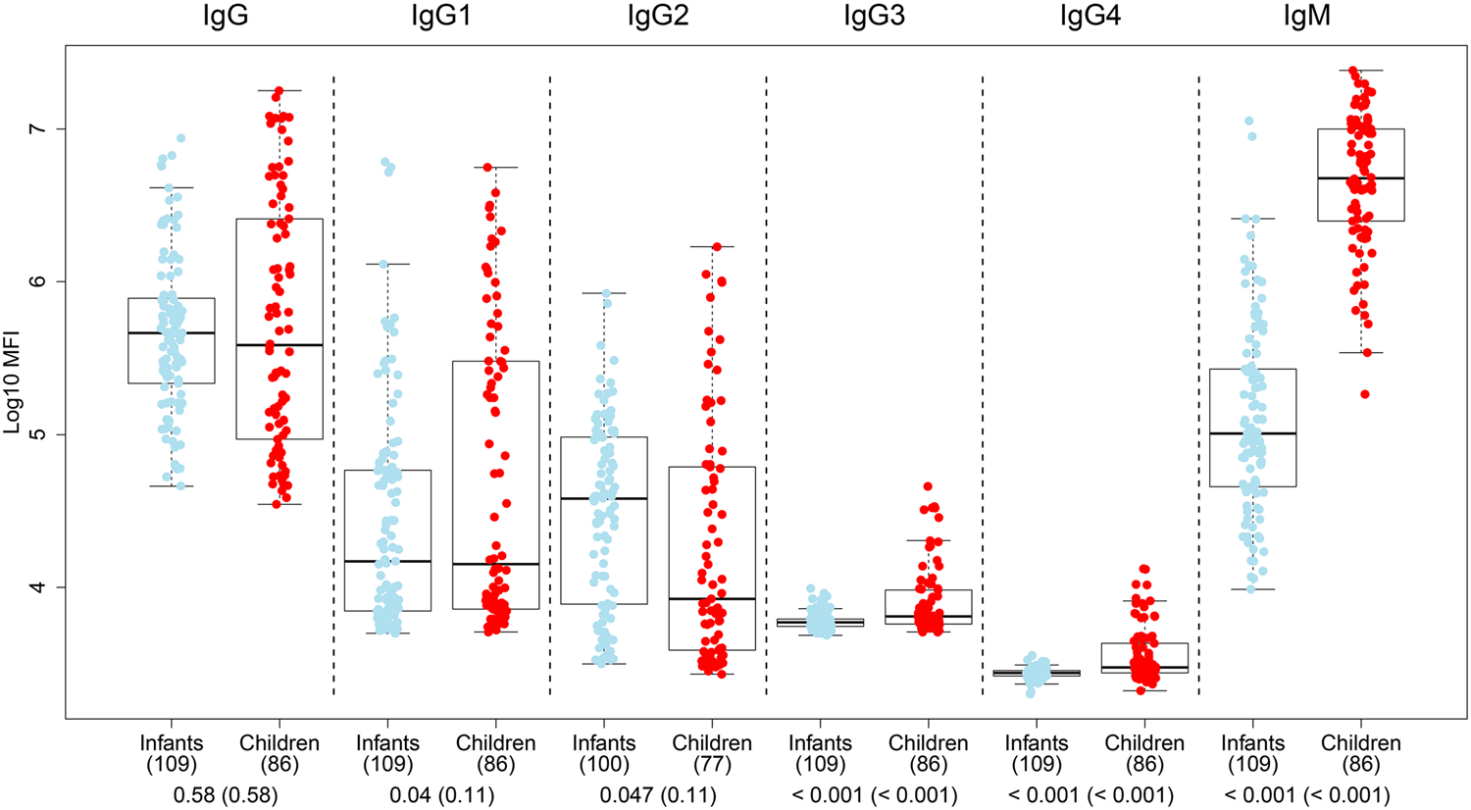
Antibody isotype and subclass responses to α-Gal by age group. Comparison of levels of IgM, IgG and IgG_1–4_ between infants (1.5–3 months old) and children (5–17 months old), both sites together. Data correspond to samples collected at recruitment (M0) from participants in the RTS,S phase 3 clinical trial. Boxplots represent the median and interquartile range. Groups were compared through t-tests and p-values were adjusted for multiple comparisons through Benjamini-Hochberg and Holm (in parenthesis).

**Figure 3:**
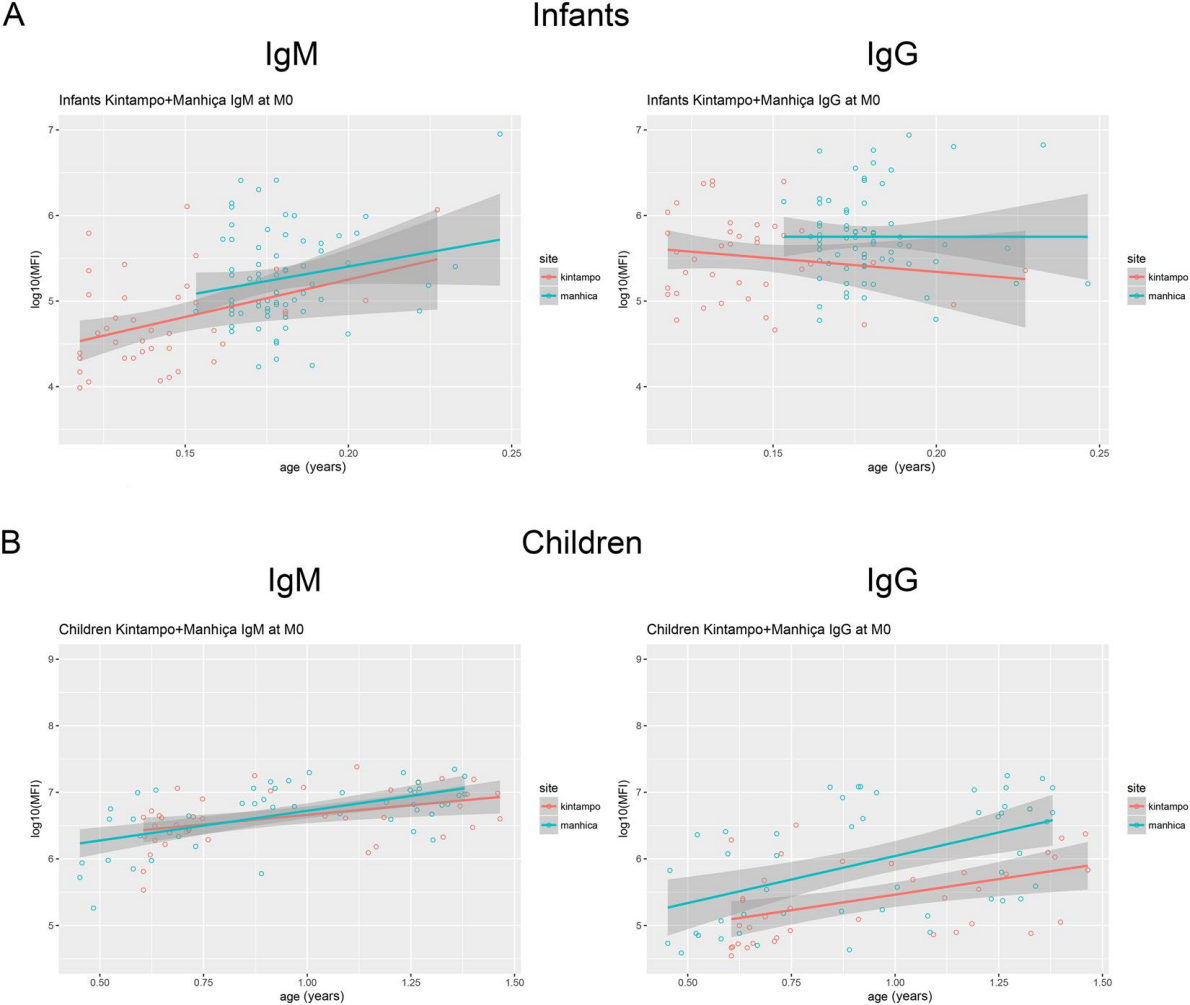
Distribution of anti-α-Gal IgG and IgM levels (log_10_MFI) as a function of age (continuous) stratified by age cohort and site (Manhiça and Kintampo). (**A**) Infants (1.5–3 months old); (**B**) children (5–17 months old). Scatter plot data correspond to samples collected at baseline (M0) from participants in the RTS,S phase 3 clinical trial.

When comparing M0 α-Gal antibody levels between the age study groups, IgMs were higher in children (5–17 months old) than in infants (1.5–3 months old) (p < 0.001), whereas total IgGs were equal between them (p = 0.58) (Fig. 2). IgG1, IgG3 and IgG4 were higher in children and IgG2 in infants, although only IgG3 and IgG4 remained significant after adjusting by multiple comparisons (p < 0.001) (Fig. 2). When analyzing the effect of continuous age on IgM and IgG levels within each age cohort, IgM showed a strong increase in infants (11 × 10^7^ MFIs/year) and a lower increase in children (3.77 MFIs/year) (Table 4 and Fig. 3). In contrast, IgG did not vary with age within the infants group, but increased 8.62 MFIs/year within the children group (Table 4 and Fig. 3). These results suggest that IgM increases from birth towards older ages, while total IgG does not increase during the initial months of life but an increase is already detected at age >5 months old. These observations were corroborated by multivariable linear regression models, showing that M3 levels of all six anti-α-Gal Ig increased with age as continuous or categorical (Table 2), but when stratifying by age group, IgM increased with age only in infants, while in children increments were observed for IgG, IgG2 and IgM (Table 3).

**Table 4:** Regression models to assess the effect of age on anti-α-Gal antibody levels and interaction with site in infants (1.5–3 months old) and children (5–17 months old) from Manhiça and Kintampo.

	**Coefficient**	**Std. Error**	**t value**	**Pr(>|t|)**
	**IgM**			
**Infants**				
(Intercept)	3.6632	0.5562	6.587	1.83E-09
Age	8.0381	3.8208	2.104	**0.0378**
Manhiça	0.3859	0.9327	0.414	0.6799
Age-site (Manhiça) interaction	−1.2675	5.6345	−0.225	0.8225
	**IgG**			
(Intercept)	5.969	0.4852	12.302	<2e-16
Age	−3.1377	3.3335	−0.941	0.349
Manhiça	−0.2149	0.8137	−0.264	0.792
Age-site (Manhiça) interaction	3.1261	4.9158	0.636	0.526
	**IgM**			
**Children**				
(Intercept)	6.0856	0.2015	30.201	<2e-16
Age	0.5762	0.2035	2.831	**0.00584**
Manhiça	−0.2545	0.2701	−0.942	0.34871
Age-site (Manhiça) interaction	0.3149	0.2721	1.158	0.25042
	**IgG**			
(Intercept)	4.5273	0.3568	12.689	<2e-16
Age	0.9355	0.3604	2.596	**0.0112**
Manhiça	0.1022	0.4782	0.214	0.8313
Age-site (Manhiça) interaction	0.4787	0.4817	0.994	0.3233

Data correspond to samples collected at baseline (M0) from participants in the RTS,S phase 3 clinical trial.

Furthermore, when comparing anti-α-Gal IgM, IgG and IgG_1–4_ subclass levels between time-points within each age group, we confirmed a significant increase of IgM from M0 to M3 in both age groups (p < 0.001) (Supplementary Fig. 2), being more marked in infants (also starting at lower basal levels) (Supplementary Fig. 3). IgG, IgG1, IgG2 and IgG3 levels decreased from M0 to M3 in infants (p < 0.001, p = 0.02, p < 0.001 and p = 0.01, respectively), and were recovered in children, with IgG levels being even higher at M3 than in M0 in this group (Supplementary Figs 2 and 3). This observation is compatible with a significant maternal transfer of anti-α-Gal IgG, IgG1, IgG2 and IgG3 to the newborn, corresponding to the levels measured in infants at M0, and a decay of these IgGs during the first months of life, evidenced by the lower M3 levels in infants. Overall, the increase of anti-α-Gal IgG and IgM levels in children compared to infants at M3 suggests a continued exposure to α-Gal.

### Effect of MTI on α-Gal antibody responses

**Figure 4:**
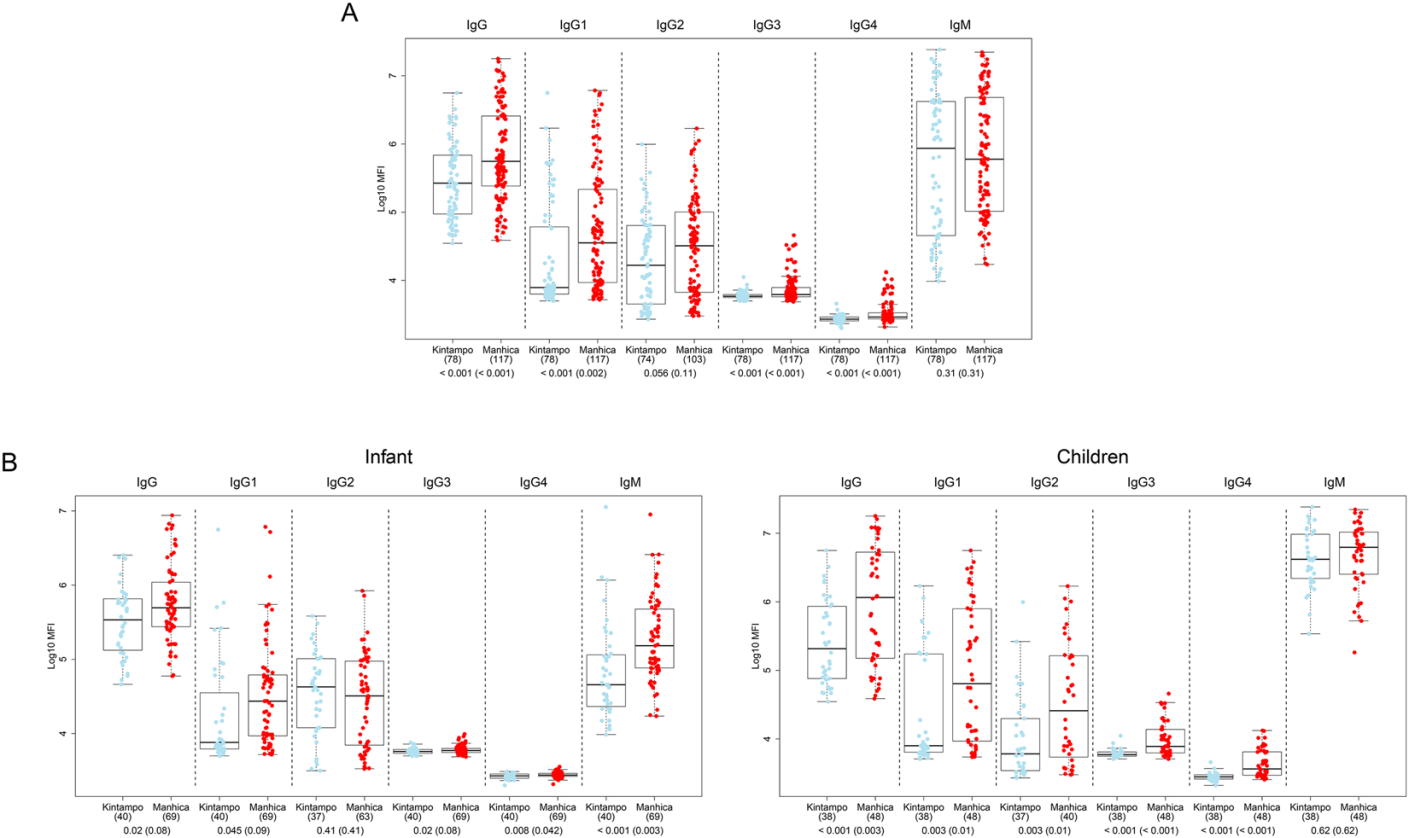
Anti-α-Gal antibody levels in Manhiça (Mozambique, low MTI) vs Kintampo (Ghana, high MTI). (**A**) Infants and children together. (**B**) Stratified by age group. Data correspond to samples collected at baseline (M0) from participants in the RTS,S phase 3 clinical trial. Boxplots represent the median and interquartile range. Groups were compared through t-tests and p-values were adjusted for multiple comparisons through Benjamini-Hochberg and Holm (in parenthesis). Infant: 1.5–3 months; Children: 5–17 months.

IgG, IgG1, IgG3 and IgG4 levels to α-Gal were significantly higher in Manhiça (low MTI) than Kintampo (high MTI) (p < 0.001, p = 0.002, p < 0.001 and p < 0.001, respectively) (Fig. 4A). When stratifying by age cohort, IgM and IgG4 were higher in infants (1.5–3 months) from Manhiça compared to Kintampo (p = 0.003 and p = 0.042, respectively), and total IgG, IgG1 and IgG3 showed a trend in the same direction (p = 0.08, p = 0.09 and p = 0.08, respectively) (Fig. 4B). Similarly, IgG, IgG1, IgG2, IgG3 and IgG4 were higher in children from Manhiça compared to Kintampo (p = 0.003, p = 0.01, p = 0.01, p < 0.001 and p < 0.001, respectively), but IgM did not show differences in this age group (Fig. 4B). Multivariable linear regression models also showed that IgG, IgG1, IgG3, IgG4 and IgM levels were higher in Manhiça compared to Kintampo (Table 2), but this was mostly for children, because when stratifying by age group, in infants only IgM was higher in Manhiça than Kintampo (Table 3).

### Effect of baseline malaria exposure and maternal antibodies on α-Gal antibody responses

The intensity of exposure to *P. falciparum* at M0, as indicated by antibody surrogate markers, was positively associated with anti-α-Gal IgM levels at M3 in multivariable linear regression models (Coeff [CI]: 10.6 [5.32; 16.13] p < 0.001) (Table 2). However, this effect disappeared when stratifying by age group (Table 3), probably because of the reduction in the sample size. Regarding IgG responses, *P. falciparum* exposure was negatively associated with anti-α-Gal IgG3 levels in children (−3.76 [−6.15; −1.32] p < 0.001) (Table 3). The models also showed a negative effect of *P. falciparum* maternally-transferred IgGs on anti-α-Gal IgG levels (−7.33 [−12.39; 1.97] p = 0.03), IgG1 (−9.13 [−15.43; 2.35] p = 0.03), IgG3 (−2.89 [−4.56; −1.19] p = 0.004), IgG4 (−2.98 [−4.34; −1.6] p < 0.001) and IgM (−18.4 [−23.63; −12.82] p < 0.001) (Table 2), but when stratifying by age group, this effect only remained significant for IgM in infants (−16.68 [−23.74; −8.98] p < 0.001) (Table 3), probably due to the reduced sample size.

### Effect of α-Gal antibodies in protection against clinical malaria

**Figure 5:**
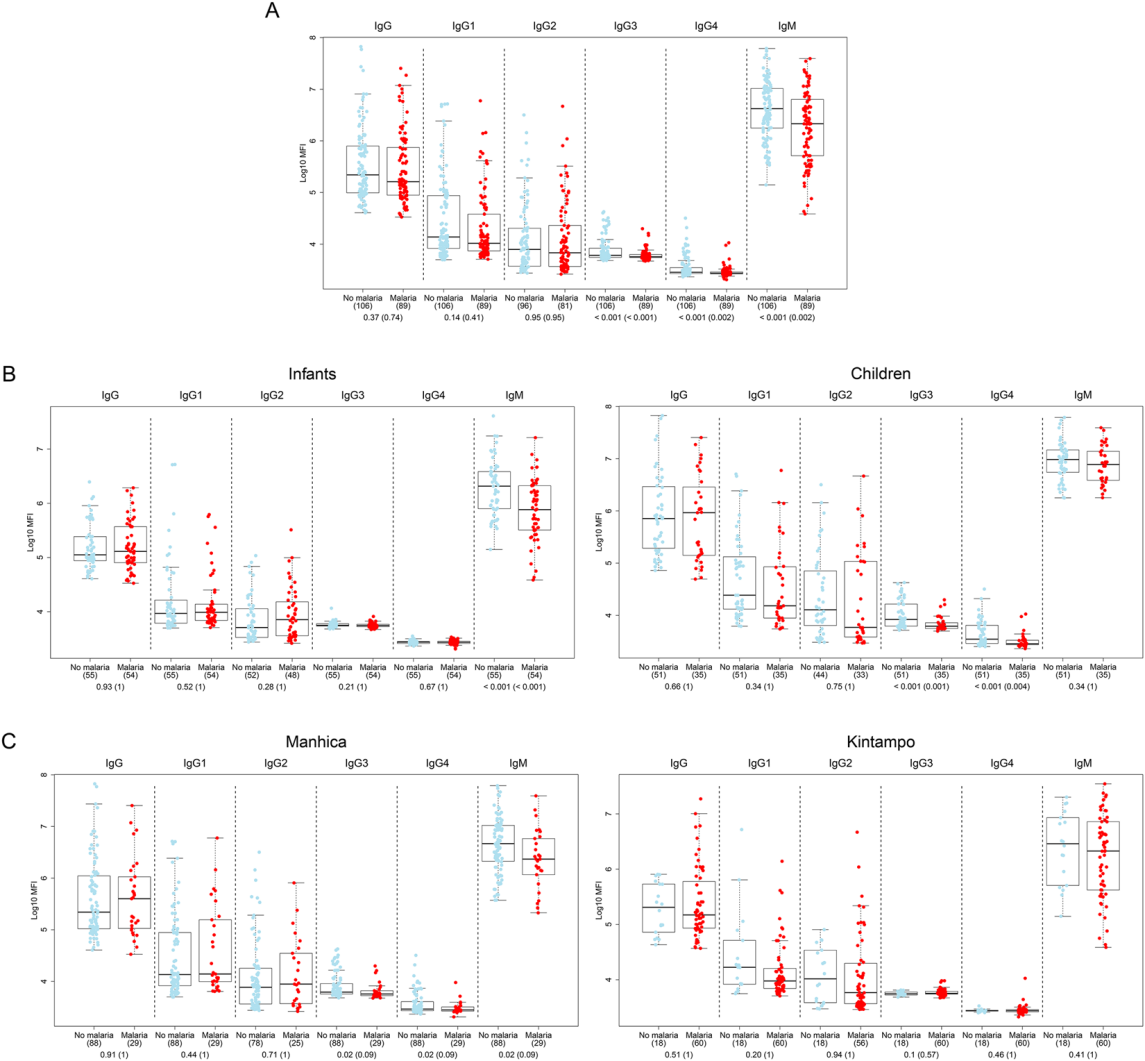
Anti-α-Gal antibody levels in cases (malaria) vs controls (no malaria). (**A**) Infants and children from both sites together. (**B**) Stratified by age group. (**C**) Stratified by site. Data correspond to samples collected at M3 (after the third vaccine dose and prior the 12 months of follow up) from children participants in the RTS,S phase 3 clinical trial. Cases were defined as children with at least one episode of clinical malaria during the 12 months of follow up. Boxplots represent the median and interquartile range. Groups were compared through t-tests and p-values were adjusted for multiple comparisons through Benjamini-Hochberg and Holm (in parenthesis). Infant: 1.5–3 months; Children: 5–17 months.

Anti-α-Gal IgM, IgG3 and IgG4 levels at M3 were higher in those subjects who did not have a clinical malaria episode over one year of follow up (p = 0.002, p < 0.001 and p = 0.002, respectively) (Fig. 5A). When stratifying by age group, IgM was higher only in infants (p < 0.001), and IgG3 and IgG4 only in children (p = 0.001 and p = 0.004) who did not subsequently develop clinical malaria (Fig. 5B). When looking at differences between cases and controls stratifying by site (but not age) (Fig. 5C), IgM, IgG3 and IgG4 were borderline significantly higher only in non-malaria controls from Manhiça (p = 0.09 for all).

Logistic regression models were fitted including the covariates significantly associated to risk of clinical malaria, like being an infant, being immunized with a comparator vaccine, being from Kintampo, having had prior malaria episodes, and having higher M0 *P. falciparum* antibodies (indicative of malaria exposure and/or maternal antibodies). Univariate models showed a protective association of anti-α-Gal IgM (OR [CI] 0.43 [0.26; 0.68], p = 0.001), IgG3 (0.02 [0; 0.18], p < 0.001) and IgG4 (0.02 [0; 0.2], p = 0.001) with clinical malaria (Table 5). Stratifying by age group, anti-α-Gal IgM correlated with less risk of clinical malaria in infants (0.24 [0.1–0.52], p < 0.001), and anti-α-Gal IgG3 (0.02 [0; 0.18], p = 0.002) and IgG4 (0.01 [0; 0.18], p = 0.003) in children (Table 5). Stratified by site, anti-α-Gal IgM had a protective role only in Manhiça (0.36 [0.15; 0.78], p = 0.055) (Supplementary Table 2). Finally, multivariable stepwise regression models adjusting by the potential cofounders revealed a significant association of anti-α-Gal IgM (0.29 [0.1; 0.77], p = 0.02) with lower risk of clinical malaria in infants; and of anti-α-Gal IgG (7.99 [1.54; 58.03], p = 0.02) with higher risk of clinical malaria in children (Table 6).

**Table 5:** Factors associated with risk of clinical malaria in univariate logistic regression models, showing odds ratios (OR) and 95% confidence intervals (CI).

Antibody	Antibody levels	Age cohort	Sex	Site	WAZ	HAZ	Hb	Exposure index	Maternal antibodies	Prior episode^†^	Season^ǂ^	Vaccine	IgG at M0	IgM at M0
	OR (CI) P-val	OR (CI) P-val	OR (CI) P-val	OR (CI) P-val	OR (CI) P-val	OR (CI) P-val	OR (CI) P-val	OR (CI) P-val	OR (CI) P-val	OR (CI) P-val	OR (CI) P-val	OR (CI) P-val	OR (CI) P-val	OR (CI) P-val
**All participants together**														
IgG	0.83 (0.55;1.24) 0.74	0.74 (0.38;1.42) 1	1.24 (0.7;2.19) 1	0.1 (0.05;0.19) <**0.001**	0.73 (0.55;0.95) 0.059	0.75 (0.57;0.96) 0.1	0.91 (0.74;1.11) 1	1.18 (1.09;1.28) <**0.001**	1.39 (1.22;1.61) <**0.001**	4.55 (1.69;14.5) **0.01**	0.66 (0.17;2.26) 1	0.64 (0.35;1.16) 0.7	0.7 (0.38;1.28) 1	0.8 (0.56;1.13) 1
**IgG1**	0.74 (0.49;1.1) 0.41	0.78 (0.43;1.43) 1	1.26 (0.71;2.23) 1	0.1 (0.05;0.19) <**0.001**	0.73 (0.56;0.95) 0.059	0.74 (0.57;0.96) 0.1	0.9 (0.73;1.11) 1	1.17 (1.08;1.27) <**0.001**	1.39 (1.22;1.6) <**0.001**	4.38 (1.62; 13.95) **0.01**	0.68 (0.17;2.36) 1	0.6 (0.33;1.1) 0.61	0.8 (0.49;1.29) 1	0.83 (0.6;1.14) 1
IgG2	1.01 (0.65;1.57) 0.95	0.79 (0.42;1.48) 1	1.48 (0.82;2.7) 1	0.1 (0.05;0.2) <**0.001**	0.7 (0.52;0.92) **0.047**	0.73 (0.55;0.94) 0.09	0.89 (0.72;1.09) 1	1.16 (1.07;1.26) <**0.001**	1.39 (1.22;1.61) <**0.001**	3.8 (1.38;12.25) **0.02**	0.49 (0.1;1.82) 1	0.66 (0.35;1.23) 0.7	0.71 (0.39;1.26) 1	0.84 (0.6;1.16) 1
IgG3	0.02 (0;0.18) <**0.001**	1.34 (0.69;2.65) 1	1.24 (0.69;2.24) 1	0.12 (0.06;0.23) <**0.001**	0.71 (0.52;0.93) 0.059	0.76 (0.58;0.99) 0.1	0.87 (0.7;1.08) 1	1.18 (1.09; 1.28) <**0.001**	1.34 (1.18;1.55) <**0.001**	3.97 (1.44;13.02) **0.02**	0.57 (0.14;2) 1	0.64 (0.34;1.19) 0.7	0.87 (0.54;1.4) 1	1.03 (0.73;1.45) 1
IgG4	0.02 (0;0.2) **0.001**	1.21 (0.63;2.36) 1	1.18 (0.66;2.11) 1	0.11 (0.06;0.22) <**0.001**	0.72 (0.54;0.95) 0.059	0.76 (0.58;0.98) 0.1	0.87 (0.7;1.07) 1	1.17 (1.09;1.28) <**0.001**	1.35 (1.18;1.56) <**0.001**	3.99 (1.44;13.08) **0.02**	0.53 (0.13;1.84) 1	0.64 (0.34;1.18) 0.7	0.82 (0.52;1.3) 1	1 (0.71;1.4) 1
IgM	0.43 (0.26;0.68) **0.001**	1.87 (0.86;4.23) 0.7	1.07 (0.59;1.93) 1	0.11 (0.05;0.21) <**0.001**	0.65 (0.48;0.86) **0.01**	0.75 (0.57;0.98) 0.1	0.84 (0.67;1.04) 0.6	1.27 (1.16;1.4) <**0.001**	1.32 (1.15;1.53) <**0.001**	4.43 (1.6;14.39) **0.01**	0.47 (0.12;1.68) 1	0.65 (0.35;1.2) 0.7	0.82 (0.51;1.29) 1	1.44 (0.91;2.33) 0.7
		**Antibody levels**	**Sex**	**Site**	**WAZ**	**HAZ**	**Hb**	**Exposure to malaria**	**Maternal antibodies**	Prior episode^†^	Season^ǂ^	**vaccine**	**IgG at M0**	**IgM at M0**
		**OR (CI) P-val**	**OR (CI) P-val**	**OR (CI) P-val**	**OR (CI) P-val**	**OR (CI) P-val**	**OR (CI) P-val**	**OR (CI) P-val**	**OR (CI) P-val**	**OR (CI) P-val**	**OR (CI) P-val**	**OR (CI) P-val**	**OR (CI) P-val**	**OR (CI) P-val**
**Infants**														
IgG		1.04 (0.43;2.51) 1	1.31 (0.61;2.81) 1	0.13 (0.05;0.32) <**0.001**	0.84 (0.58;1.22) 1	0.72 (0.5;1) 0.28	1.12 (0.81;1.58) 1	1.16 (1.03;1.33) 0.07	1.35 (1.16;1.61) <**0.001**	4.61 (1.09;31.58) 0.22	0.53 (0.13;1.91) 1	0.89 (0.4;1.98) 1	0.98 (0.38;2.53) 1	0.69 (0.36;1.26) 1
IgG1		0.81 (0.41;1.52) 1	1.29 (0.61;2.77) 1	0.14 (0.06;0.35) <**0.001**	0.84 (0.57;1.21) 1	0.72 (0.5;1) 0.28	1.11 (0.79;1.57) 1	1.16 (1.03; 1.33) 0.07	1.35 (1.15;1.61) <**0.001**	4.45 (1.04;30.73) 0.22	0.58 (0.14;2.08) 1	0.86 (0.38;1.92) 1	0.99 (0.47;2.1) 1	0.68 (0.36;1.26) 1
IgG2		1.65 (0.68;4.17) 1	1.83 (0.82;4.15) 0.83	0.15 (0.06;0.38) <**0.001**	0.77 (0.52;1.13) 0.92	0.71 (0.49;1) 0.28	1.06 (0.76;1.51) 1	1.16 (1.03;1.34) 0.07	1.38 (1.17;1.66) <**0.001**	3.19 (0.67;22.94) 0.22	0.37 (0.07;1.46) 0.95	0.91 (0.39;2.13) 1	1.01 (0.42;2.41) 1	0.74 (0.37;1.44) 1
IgG3		0.01 (0;13.86) 1	1.41 (0.65;3.06) 1	0.15 (0.06;0.36) <**0.001**	0.84 (0.57;1.22) 1	0.74 (0.52;1.03) 0.28	1.09 (0.78;1.54) 1	1.16 (1.03;1.33) 0.07	1.35 (1.15;1.59) <**0.001**	4.39 (1.03;30.19) 0.22	0.64 (0.15;2.36) 1	0.94 (0.42;2.1) 1	1.07 (0.5;2.28) 1	0.68 (0.36;1.24) 1
IgG4		0.09 (0;4636.2) 1	1.28 (0.6;2.75) 1	0.14 (0.05;0.34) <**0.001**	0.83 (0.56;1.2) 1	0.72 (0.51;1) 0.28	1.12 (0.8;1.58) 1	1.16 (1.03;1.33) 0.07	1.37 (1.16;1.63) <**0.001**	4.58 (1.06;31.87) 0.22	0.55 (0.14;1.96) 1	0.92 (0.41;2.04) 1	1.01 (0.48;2.13) 1	0.69 (0.36;1.29) 1
IgM		0.24 (0.1;0.52) <**0.001**	0.87 (0.37;1.98) 1	0.22 (0.08;0.57) **0.002**	0.74 (0.48;1.1) 0.82	0.71 (0.48;1.01) 0.28	1.02 (0.71;1.46) 1	1.24 (1.08;1.44) **0.009**	1.26 (1.07;1.5) **0.006**	3.63 (0.79;26) 0.22	0.54 (0.13;2.02) 1	1.04 (0.44;2.46) 1	1.25 (0.56;2.82) 1	1.08 (0.54;2.23) 1
			**Antibody levels**	**Sex**	**Site**	**WAS**	**HAZ**	**Hb**	**Exposure to malaria**	**Maternal antibodies**	**Prior episode** ^**†**^	**vaccine**	**IgG at M0**	**IgM at M0**
			**OR (CI) P-val**	**OR (CI) P-val**	**OR (CI) P-val**	**OR (CI) P-val**	**OR (CI) P-val**	**OR (CI) P-val**	**OR (CI) P-val**	**OR (CI) P-val**	**OR (CI) P-val**	**OR (CI) P-val**	**OR (CI) P-val**	**OR (CI) P-val**
**Children**														
IgG			0.88 (0.5;1.53) 1	1.16 (0.49;2.78) 1	0.02 (0;0.09) <**0.001**	0.59 (0.38;0.88) **0.043**	0.79 (0.52;1.18) 1	0.71 (0.52;0.96) 0.14	1.29 (1.14;1.49) <**0.001**	1.6 (1.21;2.31) **0.001**	4.78 (1.26;23.32) 0.12	0.4 (0.16;1) 0.25	0.23 (0.07;0.7) **0.05**	1.27 (0.41;4.09) 1
IgG1			0.76 (0.42;1.32) 1	1.18 (0.5;2.84) 1	0.03 (0.01;0.11) <**0.001**	0.6 (0.38;0.89) **0.043**	0.79 (0.52;1.17) 1	0.73 (0.53;0.97) 0.15	1.29 (1.14;1.49) <**0.001**	1.6 (1.22;2.31) **0.001**	4.89 (1.28;24.2) 0.12	0.39 (0.15;0.98) 0.25	0.55 (0.24;1.19) 0.5	1.25 (0.45;3.62) 1
IgG2			0.91 (0.52;1.57) 1	1.09 (0.44;2.72) 1	0.04 (0.01;0.12) <**0.001**	0.59 (0.37;0.9) **0.043**	0.75 (0.48;1.13) 1	0.73 (0.53;0.97) 0.15	1.28 (1.13;1.49) <**0.001**	1.54 (1.18;2.22) **0.002**	4.34 (1.13;21.36) 0.12	0.39 (0.15;1) 0.25	0.46 (0.18;1.11) 0.42	1.31 (0.44;4.03) 1
IgG3			0.02 (0;0.18) **0.002**	1.11 (0.44;2.8) 1	0.04 (0.01;0.15) <**0.001**	0.56 (0.34;0.88) **0.043**	0.78 (0.51;1.19) 1	0.74 (0.53;1) 0.15	1.24 (1.1;1.44) <**0.001**	1.51 (1.13;2.26) **0.007**	3.41 (0.83;17.99) 0.15	0.36 (0.13;0.97) 0.25	0.78 (0.42;1.46) 0.61	1.44 (0.49;4.49) 1
IgG4			0.01 (0;0.18) **0.003**	1.06 (0.42; 2.66) 1	0.05 (0.01;0.16) <**0.001**	0.61 (0.38;0.93) **0.043**	0.81 (0.53;1.22) 1	0.72 (0.51;0.97) 0.15	1.25 (1.1;1.45) <**0.001**	1.5 (1.11;2.25) **0.007**	3.65 (0.88;19.45) 0.15	0.36 (0.13;0.97) 0.25	0.73 (0.4;1.32) 0.61	1.25 (0.45;3.64) 1
IgM			0.55 (0.16;1.82) 1	1.18 (0.5; 2.84) 1	0.04 (0.01;0.13) <**0.001**	0.58 (0.36;0.86) **0.041**	0.79 (0.52;1.17) 1	0.73 (0.53;0.97) 0.15	1.29 (1.14;1.49) <**0.001**	1.72 (1.26;2.62) <**0.001**	4.54 (1.19;22.19) 0.12	0.4 (0.15;0.99) 0.25	0.64 (0.35;1.13) 0.5	1.68 (0.51;6.44) 1

Data from the phase 3 trial participants, including anti-α-Gal antibody data at M3 and covariates. The analysis was performed for all participants together and stratifying by age group (infants and children). Results show those factors that affect the risk of clinical malaria when anti-α-Gal antibodies are taken into account. P-values were adjusted for multiple comparisons through Benjamini-Hochberg and Holm, those significant are in bold.

Age cohort (children vs infants). Sex (male vs female). Site (Manhiça vs Kintampo). WAZ (Weight-for-Age Z-score). HAZ (Height-for-Age Z-score). Hb (Baseline hemoglobin (g/dL). Exposure index (baseline anti-*P. falciparum* exposure IgM levels). Maternal index (baseline maternally transferred antibodies). ^†^Malaria episode between month 0 and month 3 (yes vs no). ^ǂ^Malaria transmission season at month 3 sample collection (low vs high). Vaccine (RTS,S vs comparator).

**Table 6:** Association between anti-α-Gal antibody levels and risk of clinical malaria in multivariable logistic regression models.

Antibody	All subjects together		Infants		Children	
	OR (CI) p-val	Covariates*	OR (CI) p-val	Covariates*	OR (CI) p-val	Covariates*
IgG	1.86 (1;3.5) 0.051	Age, site, exposure		Site, exposure	7.99 (1.54;58.03) 0.02	Site, vaccine
IgG1		Age, site, exposure		Site, exposure		Site, vaccine
IgG2		Age, site, exposure		Site, exposure		Site, vaccine
IgG3		Age, site, exposure		Site, exposure		Site, vaccine
IgG4		Age, site, exposure		Site, exposure		Site, vaccine
IgM	0.38 (0.2;0.71) 0.003	Site, exposure	0.29 (0.1;0.77) 0.02	Site, exposure		Site, vaccine

Data from phase 3 trial including all individuals together and stratified by age group, fitted including anti-α-Gal antibody data at M3 and adjusting by significant variables in univariate models to remove potential cofounding effects in the associations. P-values were adjusted for multiple comparisons. Data presented correspond to variables that were statistically significant.

^*^Covariates that in the multivariable analyses (backward and forward stepwise algorithms combined to obtain the model with the minimum akaike information criterion) were statistically significant. Age (Infants vs children); Site (Manhiça vs Kintampo); Exposure (malaria exposure antibody index); Vaccine (RTS,S vs comparator).

## Discusion

We have assessed the IgM, IgG and IgG_1–4_ responses to α-Gal in children of different ages from two different African countries. Results show that anti-α-Gal IgM and IgG responses vary mainly depending on the age of the child and the location, but other factors like level of malaria exposure and maternally-transferred antibodies also affect them. Importantly, our data indicates that the magnitude of IgM responses to α-Gal is associated to protection against malaria, especially in the first months of life, while IgG levels may correlate with malaria risk. Our findings also point towards a possible protective role of anti-α-Gal IgG3 and IgG4 that needs to be better addressed in larger studies. Since antibodies against α-Gal are usually measured in Caucasian adults, and prior data on their levels in childhood are incomplete or even inexistent in African children^19,27,28^, our study provides novel and relevant information on anti-α-Gal antibody responses that are putative targets of immunity against several infectious diseases.

First, we provide additional insight into the age pattern of serological responses to this glycan. The anti-α-Gal IgM response in infants age 1.5 to 3 months started at very low levels but showed a rapid increase during the first months of life, reaching higher levels than IgG. This result is similar to Hamanova *et al*.^28^ on European children, and suggests exposure to α-Gal in the neonate and maintenance of this exposure over time. However, our data show an earlier and faster increase of α-Gal antibodies in African children. Exposure to α-Gal originates in the neonate gut microbiota, which is influenced by the mode of delivery, the gestational age and the mother breast milk microbiota, which in turn is influenced by maternal health^29–31^. All these factors are expected to be different between Europeans and Africans. Moreover, recent studies show that there is a significant effect of geographical variations in human milk microbiota composition^30,32^. Thus, geographical differences in human milk microbiota and exposure to pathogenic microbes could explain the differences in the anti-α-Gal IgM responses between European and African infants, and potentially among African regions.

Here, the α-Gal IgG response was already high in infants and did not increase during the first months of life. On the contrary, it tended to decrease, as evidenced when comparing Ig levels between M0 and M3 in this age group. On the other hand, in children (5 to 17 months old) IgG levels at M0 were similar to levels at M0 in infants, and increased towards M3. These results evidence a significant maternal transfer of anti-α-Gal IgG to the newborn, and a decay of this IgG during the first months of life, followed by an early and rapid increase. This suggests again a continued exposure to the glycan. These results are also similar to reports in European children^28^, although our data also suggest an earlier and faster increase of anti-α-Gal IgGs in African children compared to Europeans.

Overall, IgM and IgG to-α-Gal increased with age, however IgM reached higher levels than IgG, being the predominant response in children. This result is in agreement with previous works by Yilmaz *et al*. on subjects from 3 months to 25 years of age in Mali^17^. In that study, this observation was interpreted as indicative of *P. falciparum* infection failing to induce class switch of the anti-α-Gal Ig antibody response. However a higher IgM response than its correspondent IgG response is also observed against other polysaccharidae antigens, such as *Streptococcus pneumoniae*^33^, suggesting that overall the rate of IgM/IgG switching is not as fast for polysaccharide antigens as for protein antigens.

Second, we analyzed the effect of malaria endemicity on the anti-α-Gal response. When comparing anti-α-Gal IgM and IgG responses between two areas of high (Kintampo) and low (Manhiça) MTI, we observed that both antibodies were higher at lower MTI. This may suggest that other exposures besides malaria may be more important for their induction. It is known that, besides *Plasmodium*^17^, other pathogenic microbes express this glycan, like the protozoan parasites *Leishmania spp*. and *Trypanosoma spp*, the Gram-negative bacteria *Salmonella spp*., and some viruses^22,23,34^. Furthermore, common commensal bacteria in the midgut microbiota such as *Escherichia spp*., *Klebsiella spp*. and *Serratia spp*. also express α-Gal^22^. Also, exposure to different types of diet in both sites could be associated to the different anti-α-Gal responses. However, another possible explanation is that malaria infection affects the immune response to α-Gal in children living in high MTI. Previous studies show that *P. falciparum* malaria impairs the antibody response to polysaccharide vaccines (glycan antigens) but not responses to protein-based and whole parasite vaccines in children with malaria^35^. Young children (<3 years old) have immunologically immature spleens, mainly due to the still ongoing development of the marginal zone (MZ) B cell subset, which is the main responsible for the IgM response to polysaccharide antigens^36,37^. During malaria infection, the anatomy of the spleen becomes disorganized, with sometimes a complete dissolution of the MZ^38,39^. Accordingly, several studies have found a reduction of peripheral MZ-like B cells in patients with malaria^40–42^, which could explain the reduction in the IgM response in children with higher malaria exposure^43^ that may affect IgM response to α-Gal. However, in spite of the higher anti-α-Gal IgM response observed in the lower MTI site, the multivariable analysis showed that recent/current exposure to *Plasmodium* was positively associated to the levels of anti-α-Gal IgM, implying that malaria infection in fact induces IgM against α-Gal. These models also showed a negative effect of maternally transferred *P. falciparum* IgGs on the anti-α-Gal IgG and IgM responses in the offspring, suggesting an interference with anti-α-Gal antibody induction in children. A negative effect of maternal antibodies has been reported in the context of immune responses to vaccines^44^.

Third, we investigated the role of α-Gal antibodies in malaria risk or protection. Remarkably, anti-α-Gal IgM levels were higher in infants who did not subsequently develop any episode of malaria. Interestingly, this association was only observed in Manhiça in site-stratified analysis. The fact that this association was only observed in infants but not in children contrasts with the results by Yilmaz *et al*., where anti-α-Gal IgMs were associated to protection in Malian children >4 years old^17^. Disparity may result from several differences between the studies: (i) different samples sizes (195 in our study vs 695 in Mali study); (ii) separate countries with different levels of α-Gal exposure due to malaria and other pathogens; (iii) different follow up times (12 months in our study vs 6 months in Mali); (iv) different age ranges of subjects (1.5 to 17 months in our study vs 4 to 25 years in Mali); and, specially, due to the different ways to detect and define clinical malaria (passive case detection [PCD] defined by fever with any parasitemia in our study vs active case detection defined by fever with parasitemia ≥2500 parasites/mL in the Mali study).

Unlike IgM, anti-α-Gal IgG levels were associated with a higher risk of malaria in children, which suggests that a higher exposure to other pathogenic microbes containing α-Gal may increase the risk of a future malaria episode by, for example, deviating the immune response and/or causing a worst clinical outcome in co-infection. This result contrasts with the recent observation by Cabezas-Cruz *et al*. of a positive correlation of anti-α-Gal IgM and IgG with the lack of *Plasmodium* infection in individuals from Senegal^45^. However disparity of results may also be due to differences between the study site, age of participants and the study design.

We also investigated for the first time IgG_1–4_ subclass responses to α-Gal and observed new associations between certain subclasses and malaria protection. Interestingly, the pattern of IgG_1–4_ subclasses to α-Gal showed predominance of IgG1 and IgG2, followed by IgG3 and IgG4. This is different to the pattern against *P. falciparum* proteins, where IgG1 and IgG3 predominate and IgG2 and IgG4 are induced at much lower levels. Higher α-Gal IgG3 and IgG4 levels may correlate with malaria protection in children, also contrasting to what has been observed against protein antigens. Previous studies consistently show that cytophilic antibodies (IgG1 and IgG3) to protein antigens correlate more often with protection from malaria disease^46–50^. However, this may be different for glycan antigens. For example, IgG4 responses predominate against *Schistosoma mansoni*^51^ with many antigenic glycans on its surface^52^, and this subclass is associated with protection against *S. haematobium*^53^. IgG4 has been shown to be a blocking and tolerance-inducing “anti-allergenic” antibody^54,55^. Therefore its protective effect could be mediated through a tolerogenic response to the *Plasmodium* infection. However, IgG3 and IgG4 associations to malaria protection were lost in multivariable analysis and further studies with larger samples sizes are needed to better address this potential protective effect.

The study had some limitations, mostly related to the fact that it was performed with samples from the RTS,S clinical trial consisting on two age cohorts, forcing some design issues, e.g., the age range and the vaccination. Second, the unfeasibility to determine the exposure to other sources of α-Gal besides malaria, including other pathogens, commensal bacteria or food, which would have been helpful to understand why anti-α-Gal responses were higher in one site vs the other. Nevertheless, the fact the children from high MTI settings showed significantly lower levels of anti-α-Gal IgG and IgM compared to children living in lower MTI settings, might be a sign of an underlying impairment of the immune response to polysaccharide antigens in the context of high MTI. These data along with the observed reduction of MZ-like B cells in chronically exposed individuals^40–42^, the documented deficient antibody response to polysaccharide vaccines in children with malaria^35^ and the higher susceptibility of these children to invasive bacterial infections by polysaccharide encapsulated bacteria (as non-typhoid salmonella and *S. pneumoniae)*^56,57^, warrant further investigation.

## Conclusions

Age and site affect the magnitude of anti-α-Gal IgM and IgG responses in African children. Levels of α-Gal IgG3, IgG4 and, particularly, IgM are associated with protection against clinical malaria, while total IgG levels correlate with malaria risk, supporting further investigations of α-Gal as a promising antigen target for future malaria vaccines.

## Materials and Methods

### Subjects and samples

Samples from African children participating in RTS,S/AS0 clinical trials were included in this analysis. First, a pilot study to assess age patterns of anti-α-Gal antibody immunogenicity in individuals age <5 years old was performed with serum samples from 104 Mozambican children of two cohorts exposed to different levels of MTI (Manhiça - low MTI, and Ilha Josina - high MTI), vaccinated with RTS,S/AS02 within a phase 2b trial^58^. This pilot was carried out to set up the α-Gal antibody assay and to confirm the age pattern of response previously described in Malian children^17^. A second larger study was performed to assess IgG subclasses and association between anti-α-Gal antibodies and malaria protection, and factors affecting it. This analysis included plasma and serum samples from 195 subjects enrolled in the RTS,S/AS01 phase 3 trial^59^ from two younger age groups (1.5–3 months [infants] and 5–17 months [children]), and two different African sites (Manhiça-Mozambique [low MTI] and Kintampo-Ghana [moderate-high MTI]), having 131 RTS,S-vaccinees and 66 comparator-vaccinees.

In both studies, samples were collected at the first study visit (recruitment, coinciding with baseline before vaccination, M0) and the second study timepoint three months later (M3, after three doses of vaccination). For the detection of clinical malaria cases (fever >37.5 °C with any parasitaemia), children were followed up by PCD starting 14 days after sample collection at M3 for the subsequent 12 months.

### Antibody Luminex assay

Antibodies against α-Gal (Galα1-3 Galβ1-4GlcNAc-R-BSA, Dextra NGP0334) were measured by quantitative suspension array technology (qSAT) using the Luminex xMAP™ technology (Luminex Corp., Austin, Texas). α-Gal was covalently coupled to MagPlex beads and these were blocked with BSA. α-Gal-coupled beads were added to 96-well μClear® flat bottom plates (Greiner Bio-One) (1,000 microspheres/well) resuspended in 50 µL of PBS, 1% BSA, 0.05% Azide pH 7.4 (PBS-BN). The multiplex antigens panel also contained beads coupled to 32 *P. falciparum* protein constructs based on pre-erythrocytic (SSP2/TRAP, CelTOS, LSA-1, EXP-1) and erythrocytic (AMA-1, EBA-140, EBA-175, MSP-1, MSP-2, MSP-3, MSP-5, MSP-6, CyRPA, P41, PfRh1, PfRh2, PfRh4, var2csa) stage antigens analyzed as markers of malaria exposure and maternally-transferred antibodies.

Fifty µL of sample, positive control (serial dilutions of the WHO reference reagent for anti-malaria human serum NIBSC code 10/198 for the IgG assays; or a pool of samples with high IgM levels against *P. falciparum* for the IgM assay)^60^, negative control (individual plasma samples from malaria naïve Spanish adults) or PBS-BN (Blanks) were added to the wells and incubated with the beads at 4 °C overnight (ON) in a shaker protected from light. Plates were washed three times with 200 µL/well of PBS-Tween 20 0.05% using a manual magnetic washer. 100 µL of biotinylated secondary antibody were added diluted in PBS-BN as described^61^: anti-human IgG (Sigma), anti-human IgM (Sigma), anti-human IgG1 (Abcam) and anti-human IgG3 (Sigma). For IgG2 and IgG4 assays, secondary antibodies added were unconjugated mouse anti-human IgG4 (Thermo Fisher) and mouse anti-human IgG2 (Thermo Fisher), respectively, followed by biotinylated goat anti-mouse IgG (Sigma) in PBS-BN. All antibody incubations were performed at room temperature (RT) for 60 min, in agitation and protected from light. Next, 100 µL of streptavidin-R-phycoerythrin (Sigma) in PBS-BN were added to all wells and incubated 30 min, at RT, in agitation and protected from light. Plates were washed as before and beads were resuspended in 100 μL/well of PBS-BN. Plates were covered protected from light and stored at 4 °C ON to be read the next day using the Luminex xMAP® 100/200 analyser, and at least 50 microspheres per analyte were acquired per sample.

Test samples were assayed at 4 dilutions for IgG (500, 5000, 50,000 and 500,000), IgG1, IgG3 (100, 1000, 10,000 and 100,000) and IgM (100, 1000, 10,000 and 50,000), and 2 dilutions for IgG2 and IgG4 (50 and 500) to ensure that at least one dilution lie in the linear range of the respective standard curve. For IgG assays, 18 to 22 serial dilutions (1:2) of the positive control starting at 1:50 were used to perform subclass-specific standard curves. For the IgM assay, 18 serial dilutions (1:2) of a pool of samples from ISGlobal repository with high IgM levels against *P. falciparum* antigens were used. Blanks were added to each plate in triplicates for quality control purposes. Sample distribution across plates was designed ensuring a balanced distribution of site, age cohort and malaria cases. Data were captured using xPonent software, and antibody levels were measured as median fluorescence intensity (MFI).

### Data analysis

#### Preprocessing

To stabilize the variance, the analysis was done on log_10_-transformed values of the MFI measurements. The positive control standard curve for each isotype/subclass-plate was estimated using the *drLumi* R package flow^62^. Standard curves were fitted in a 5-parameter logistic (5-PL) regression model, and data points were weighted by logarithmic variance. If the model did not converge, 4-PL or exponential regressions were fitted. The quality control for each plate was based on the estimation of the % coefficient of variation (CV) of the 3 blank controls. Blanks were also used to establish the antigen-isotype/subclass specific lower limits of quantification (LLOQ) and lower limits of detection (LLOD) calculated as the blanks mean +10 SD and blanks mean +3 SD, respectively^63^. The characteristics of the standard curves were visually inspected for quality control purposes. To select the sample working dilution (isotype/subclass and plate specific), an algorithm that detects the two points with the highest slope between them in the positive control sigmoidal curve was used. The slope was computed as:

$$m=\frac{log10{(MFI)}_{i}-log10{(MFI)}_{i+1}}{dilution\_facto{r}_{i}-dilution\_facto{r}_{i+1}}$$

The mean log_10_ MFI value of the two points was computed, and the nearest log_10_ MFI of the test sample and the corresponding dilution was selected. For IgG2 and IgG4 assays standard curves did not converge, then the first sample dilution was assigned. The log_10_ MFI of the selected dilution was corrected multiplying by its corresponding dilution factor. Blank background signal was not subtracted.

#### Statistical analysis

Descriptive comparisons of antibody levels between age groups, time points and sites were done by trajectory plots, boxplots representing the median and interquartile range (analyzed by t-tests), and dotplots with bars corresponding to the geometric mean and confidence intervals (CI) (analyzed by the Mann Whitney t-test). The effect of age was also evaluated through scatterplots and regression models and assessing its interaction with site.

The analysis of factors affecting levels of anti-α-Gal Ig at M3 was performed using data from children participating in the RTS,S phase 3 trial and applying multivariable linear regression models (Coefficient, 95% CI, p values). The predictors assessed were: age as continuous variable (weeks), age cohort (children vs infants), sex (male vs female), site (Manhiça vs Kintampo), baseline weight for age Z score (WAZ) and height for age Z score (HAZ), baseline hemoglobin levels (Hb), malaria episodes prior to M3 (yes vs no), malaria transmission season (low vs high), vaccination (RTS,S vs comparator), baseline α-Gal IgG levels, baseline α-Gal IgM levels, level of malaria exposure and maternally-transferred malaria antibodies. To define a *P. falciparum* exposure index, we selected 28 protein antigens in which IgM responses were M3 > M0 and thus acquired with age (e.g. children > infants) and exposure (e.g. Kintampo > Manhiça) (data not shown). Principal component analysis (PCA) was performed to construct the corresponding variables, and the first component (PC1) that explained 63% of the variability was selected to be used as a variable in the models. To define a *P. falciparum* maternal antibody index in subjects <10 months of age, we selected 17 antigens including two VAR2CSA pregnancy-specific antigen constructs which IgG responses were M0 > M3 and thus declined with age (e.g. infants > children) and were higher in infants from the high MTI site (e.g. Kintampo > Manhiça) (data not shown). We selected the first component that explained 54% of the variability and used that as a variable in the models.

The analysis of the association between anti-α-Gal antibody levels and clinical malaria was based on a case-control design. Univariate logistic regression models (odds ratio [OR], 95% CI, p values) with α-Gal antibody data at M3 as main predictor, including other covariates (same as above) and their interactions, were fitted to identify factors that affected malaria risk when α-Gal antibodies were taken into account. Covariates that were significant in the univariate models were included in the stepwise (forward and backward) multivariable models to remove potential cofounding effects. P-values were adjusted for multiple testing through Benjamini-Hochberg or Holm, depending on the analysis. None of the interactions were significant after adjusting for multiple comparisons, therefore they are not reported in the tables. All models were also performed stratifying by age group, by site, and by age and site at the same time. Significance was defined at the p < 0.05 level and analyses were performed with R.

### Data availability

All data generated or analyzed during this study are included in this published article (and its Supplementary Information files).

### Ethics Statement

All methods were performed in accordance with the relevant guidelines and regulations. Approval for the study protocol was obtained from the Ethical Committee of the Hospital Clínic in Barcelona (CEIC, Spain), the National Health and Bioethics Committee (CNBS, Mozambique), and the Ghana Health Service Ethical Review Committee (GHSERC, Ghana). Written informed consent was obtained from parents or guardians of participating children in accordance with the Declaration of Helsinki.

## Electronic supplementary material

Supplementary Information
