# Supplementary material for: Antibody responses to α-Gal in African children vary with age and site and are associated with malaria protection
Source: Sci Rep. 2018 Jul 3;8:9999. doi: 10.1038/s41598-018-28325-w (PMC6030195; doi:10.1038/s41598-018-28325-w)
Supplement: Supplementary file 1 — Supplementary Information [file 41598_2018_28325_MOESM1_ESM.pdf]

Antibody responses to α-Gal in African children vary with age and site and are associated with malaria protection

Ruth Aguilar[1], Itziar Ubillos[1], Marta Vidal[1], Núria Balanza[1], Núria Crespo[1], Alfons Jiménez[1], Augusto Nhabomba[2], Chenjerai Jairoce[2], David Dosoo[3], Ben Gyan[3], Aintzane Ayestaran[1], Hèctor Sanz[1], Joseph J. Campo[1], Gloria P. Gómez-Pérez[1], Luis Izquierdo[1*], Carlota Dobaño[1*]

Affiliations

[1] ISGlobal, Hospital Clínic - Universitat de Barcelona, Carrer Rosselló 153 (CEK building), E-08036 Barcelona, Catalonia, Spain.

[2] Centro de Investigação em Saúde de Manhiça (CISM), Rua 12, Cambeve, Vila de Manhiça, CP 1929, Maputo, Mozambique.

[3] Kintampo Health Research Center, Kintampo, Ghana.

* Corresponding authors: Luis Izquierdo (luis.izquierdo@isglobal.org) and Carlota Dobaño (carlota.dobano@isglobal.org)

SUPPLEMENTARY MATERIAL

Supplementary Figure 1. Scatter plots with lines representing the distribution of anti-α-Gal IgG and IgM levels in children between 1 and 4 years old stratified by site (Manhiça and Ilha Josina). Data correspond to samples collected at baseline (M0) from children participants in the RTS,S Phase 2b clinical trial.

IgM

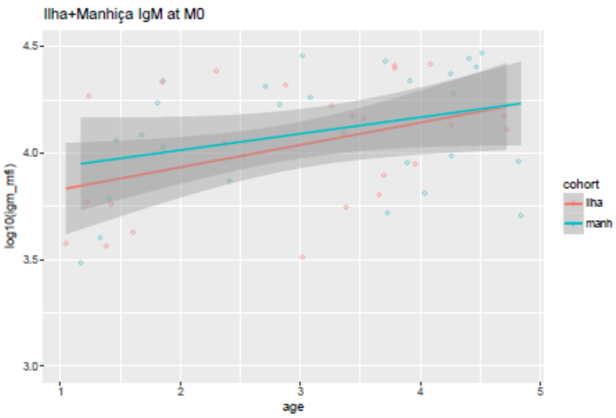

IgG

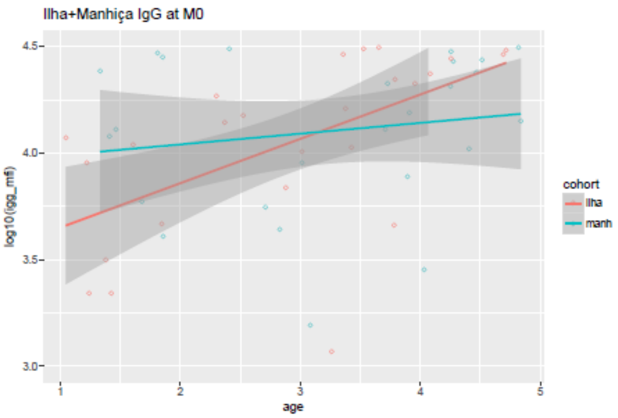

Supplementary Figure 2. Anti-α-Gal antibody levels by time-points in infants and children. Data correspond to samples collected at baseline (M0) and 3 months after vaccination (M3) from participants in the RTS,S phase 3 clinical trial from Manhiça and Kintampo (both sites together). Boxplots represent the median and interquartile range. Groups were compared through t-tests and p-values were adjusted for multiple comparisons through Benjamini-Hochberg and Holm (in parenthesis). Infants: 1.5-3 months; Children: 5-17 months.

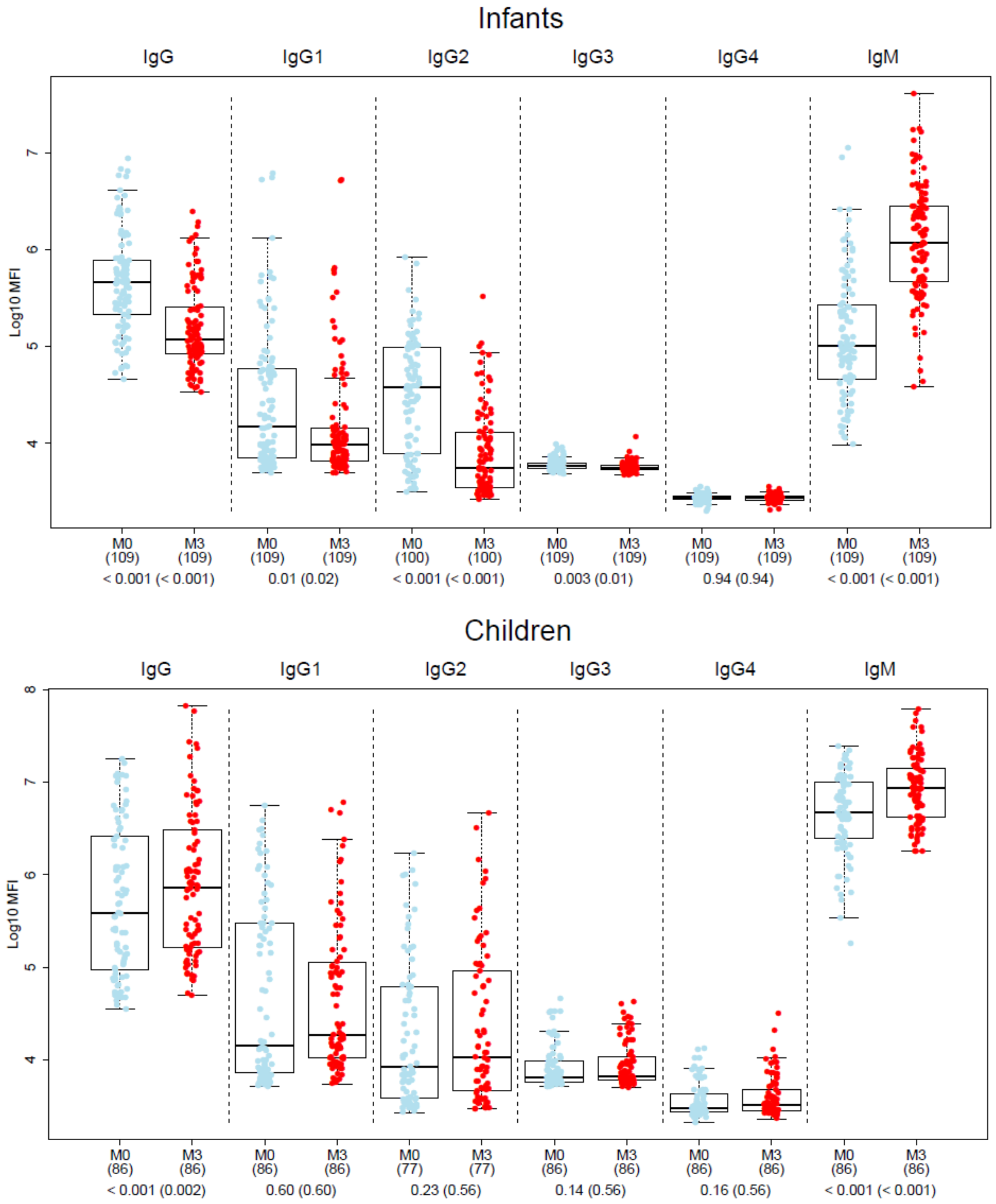

Supplementary Figure 3. Change in anti-α-Gal IgG and IgM levels between time-points in infants and children. A) Both sites together. **B)** Stratified by site. Trajectory plot data correspond to samples collected at baseline (M0) and 3 months after vaccination (M3) from participants in the RTS,S phase 3 clinical trial.

A

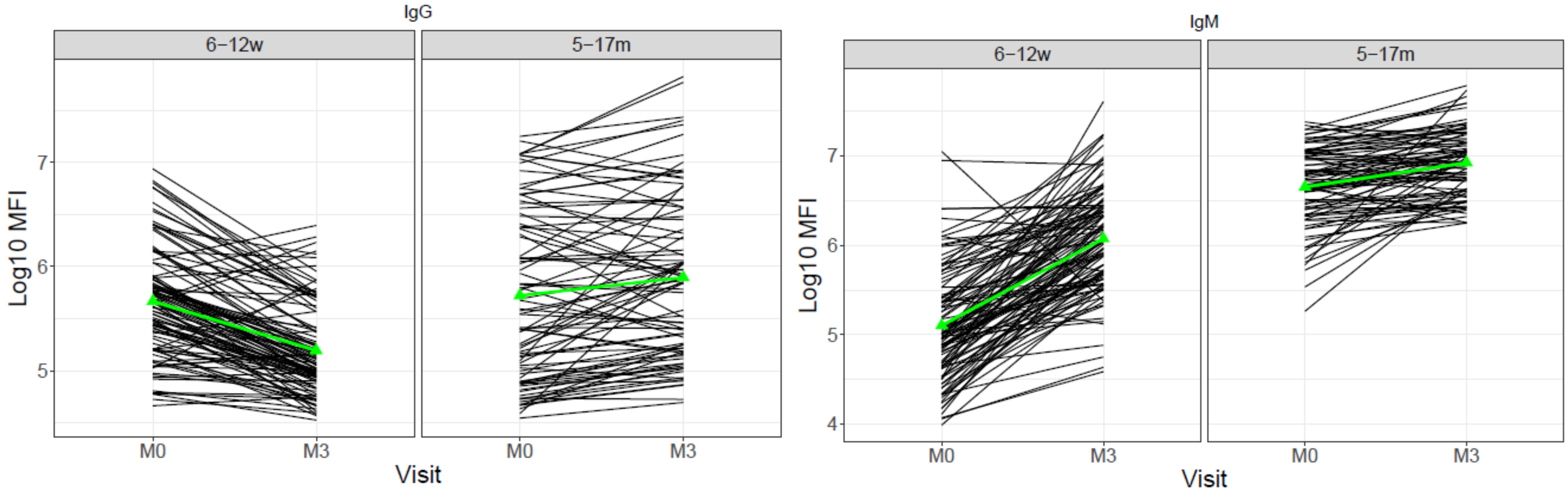

B

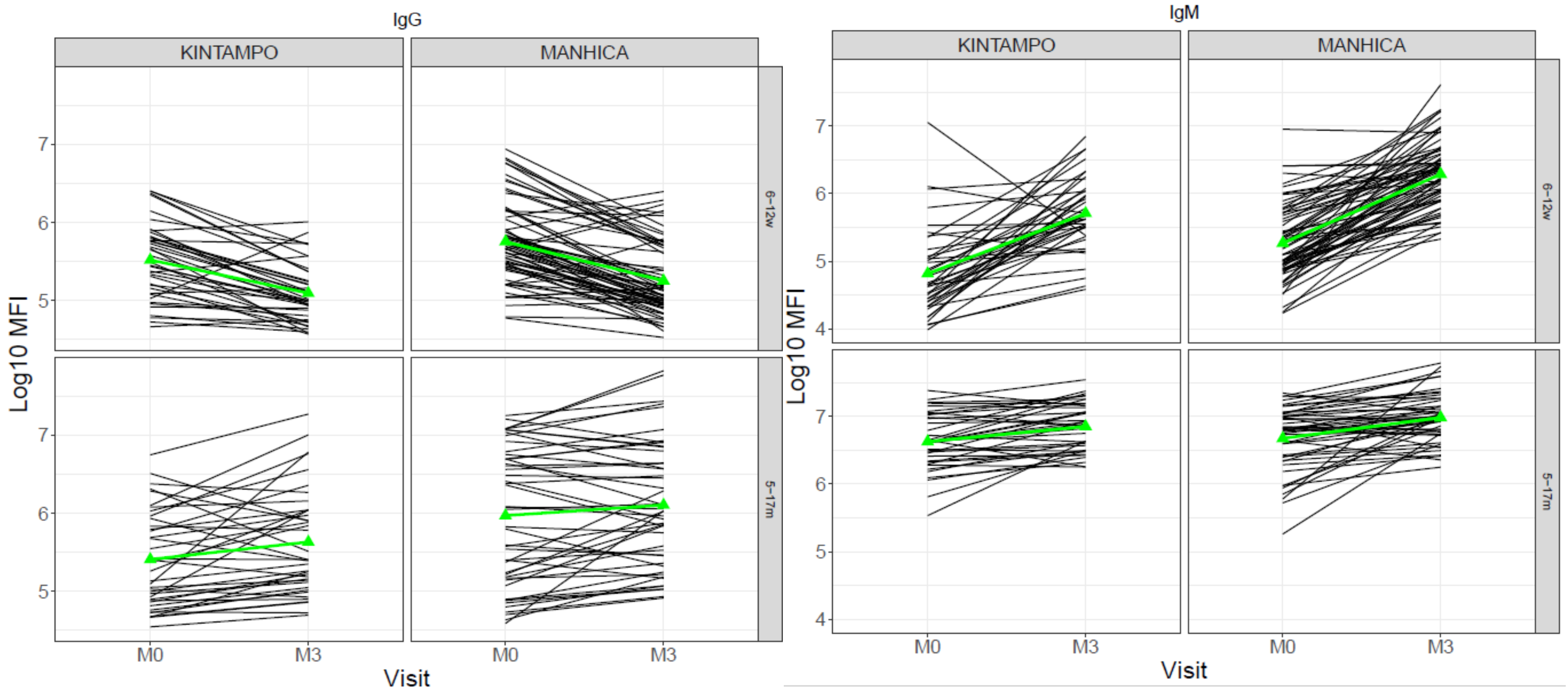
IgG
KINTAMPO
MANHICA
6−12w
5−17m
Log10 MFI
7
6
5
M0
M3
Visit
IgM
KINTAMPO
MANHICA
6−12w
5−17m
Log10 MFI
7
6
5
4
M0
M3
Visit

Supplementary Table 1. Effect of RTS,S/AS01E vaccination on anti-α-Gal antibody responses at M3 by site and age cohort. Interpretation in MFI scale (percent change for a units change in the predictor). P-Adj for multiple testing was done using Holm method.

	vaccine (RTS,S vs comparator)											
	Infants						Children					
	Kintampo			Manhiça			Kintampo			Manhiça		
**Antibody**	**Coef(CI)**	**P**	**P-Adj**	**Coef(CI)**	**P**	**P-Adj**	**Coef(CI)**	**P**	**P-Adj**	**Coef(CI)**	**P**	**P-Adj**
**IgG**	69.55 (-3.89;199.13)	0.07	0.34	-4.48 (-44.03;63.03)	0.86	1	-3.58 (-67.05;182.18)	0.95	1	-32.56 (-78.94;116.01)	0.5	1
**IgG1**	-28.55 (-70.03;70.37)	0.44	1	-40.74 (-71.2;21.96)	0.15	0.92	29.41 (-48.24;223.54)	0.57	1	-52.11 (-86.43;68.98)	0.25	1
**IgG2**	128.92 (5.83;395.17)	0.04	0.22	-3.21 (-42.22;62.14)	0.9	1	-12.16 (-75.78;218.54)	0.84	1	-45.48 (-85.23;101.23)	0.35	1
**IgG3**	104.34 (-61.76;992.08)	0.39	1	-54.06 (-87.99;75.75)	0.25	1	7.46 (-79.28;457.15)	0.93	1	-10.28 (-53.39;72.72)	0.74	1
**IgG4**	2.63 (-1.62;7.06)	0.22	0.89	1.97 (-2.53;6.68)	0.39	1	-0.77 (-16.04;17.27)	0.93	1	2.53 (-27.76;45.54)	0.89	1
**IgM**	28.83 (-44.07;196.77)	0.54	1	28.01 (-28.5;129.19)	0.4	1	-31.92 (-60.81;18.26)	0.17	1	24.61 (-26.32;110.77)	0.4	1

CI = confidence interval

Supplementary Table 2. Factors associated to the risk of clinical malaria in univariate logistic regression models stratified by site. Anti-α-Gal antibody data at M3 and covariates from phase 3 participants. Results show those factors that affected the risk of clinical malaria when anti-α-Gal antibodies were taken into account. P-values were adjusted for multiple comparisons through Benjamini-Hochberg and Holm; those significant are in bold.

Manhiça

Antibody	Antibody levels	Age cohort	Sex	WAZ	HAZ	Hb	Exposure to malaria	Maternal antibodies	Prior episode †	Vaccine	IgG at M0	IgM at M0
	OR (CI) P-val	OR (CI) P-val	OR (CI) P-val	OR (CI) P-val	OR (CI) P-val	OR (CI) P-val	OR (CI) P-val	OR (CI) P-val	OR (CI) P-val	OR (CI) P-val	OR (CI) P-val	OR (CI) P-val
**IgG**	1.03 (0.58;1.79) 1	0.12 (0.03;0.43) **0.004**	1.12 (0.48;2.63) 1	0.72 (0.47;1.08) 0.36	0.68 (0.45;1) 0.23	0.81 (0.53;1.25) 1	1.18 (1.03;1.37) 0.056	1.24 (1.02;1.53) 0.14	0 (NA;2.083e +63) 1	0.73 (0.3;1.78) 1	1.81 (0.72;4.9) 0.89	0.54 (0.29;0.98) 0.25
**IgG1**	1.24 (0.73;2.06) 1	0.18 (0.05;0.52) **0.006**	1.13 (0.48;2.64) 1	0.72 (0.47;1.08) 0.36	0.69 (0.45;1.01) 0.23	0.81 (0.52;1.24) 1	1.17 (1.02;1.36) 0.056	1.25 (1.02;1.54) 0.14	0 (NA;1.800e +63) 1	0.76 (0.32;1.89) 1	1.24 (0.61;2.55) 0.89	0.59 (0.34;0.99) 0.25
**IgG2**	1.13 (0.57;2.1) 1	0.17 (0.04;0.59) **0.02**	1.45 (0.58;3.69) 1	0.68 (0.44;1.04) 0.36	0.64 (0.41;0.97) 0.23	0.81 (0.5;1.28) 1	1.17 (1.02;1.37) 0.056	1.29 (1.05;1.63) 0.1	0 (NA;1.325e +108) 1	0.72 (0.28;1.86) 1	1.71 (0.71;4.27) 0.89	0.6 (0.32;1.09) 0.38
**IgG3**	0.08 (0;0.83) 0.16	0.35 (0.08;1.22) 0.24	1.08 (0.46;2.56) 1	0.67 (0.42;1.04) 0.36	0.69 (0.45;1.01) 0.23	0.72 (0.44;1.15) 1	1.19 (1.04;1.39) **0.045**	1.18 (0.97;1.47) 0.3	0 (NA;1.396e +105) 1	0.74 (0.31;1.84) 1	1.59 (0.81;3.21) 0.89	0.84 (0.46;1.5) 1
**IgG4**	0.06 (0;0.93) 0.17	0.33 (0.08;1.13) 0.24	1.01 (0.43;2.4) 1	0.68 (0.43;1.05) 0.36	0.68(0.45;1) 0.23	0.7 (0.43;1.12) 0.84	1.21 (1.05;1.41) **0.04**	1.19 (0.97;1.47) 0.3	0 (NA;1.549e +104) 1	0.74 (0.3;1.83)	1.48 (0.77;2.9) 0.89	0.83 (0.45;1.5) 41
**IgM**	0.36 (0.15;0.78) 0.055	0.44 (0.13;1.43) 0.24	0.87 (0.35;2.13) 1	0.66 (0.42;1.01) 0.34	0.67 (0.44;0.99) 0.23	0.78 (0.49;1.23) 1	1.22 (1.06;1.43) **0.03**	1.18 (0.96;1.46) 0.3	0 (NA;1.643e +63) 1	0.8 (0.33 ; 2.04) 1	1.65 (0.84;3.32) 0.89	1.09 (0.53 ; 2.35) 1

Kintampo

	Antibody levels	Age cohort	Sex	WAS	HAZ	Hb	Exposure to malaria	Maternal antibodies	Prior episode †	Vaccine	IgG at M0	IgM at M0
**Antibody**	**OR (CI) P-val**	**OR (CI) P-val**	**OR (CI) P-val**	**OR (CI) P-val**	**OR (CI) P-val**	**OR (CI) P-val**	**OR (CI) P-val**	**OR (CI) P-val**	**OR (CI) P-val**	**OR (CI) P-val**	**OR (CI) P-val**	**OR (CI) P-val**
**IgG**	1.3 (0.54;3.58) 1	0.78 (0.24;2.54) 1	1.83 (0.63;5.61) 1	1.11 (0.68;1.8) 1	1.25 (0.79;2.03) 1	1.08 (0.81;1.45) 1	1.04 (0.93;1.18) 1	1.21 (0.94;1.56) 0.69	1.82 (0.51;8.62) 1	0.41 (0.1;1.32) 0.82	0.69 (0.21 ; 2.26) 1	0.91 (0.51 ; 1.59) 1
**IgG1**	0.52 (0.22;1.2) 0.72	1.18 (0.39;3.72) 1	1.97 (0.66;6.27) 1	0.99 (0.61;1.62) 1	1.2 (0.76;1.95) 1	1.05 (0.78;1.42) 1	1.05 (0.94;1.19) 1	1.07 (0.8;1.41) 0.84	1.59 (0.43;7.61) 1	0.4 (0.1;1.32) 0.82	1.02 (0.38 ; 2.77) 1	1.14 (0.66 ; 1.99) 1
**IgG2**	0.97 (0.45;2.32) 1	1 (0.34;2.97) 1	1.95 (0.67;6.01) 1	0.99 (0.61;1.61) 1	1.2 (0.75;1.94) 1	1.06 (0.79;1.42) 1	1.05 (0.94;1.18) 1	1.17 (0.92;1.5) 0.84	1.67 (0.46;7.98) 1	0.44 (0.11; .42) 0.82	0.95 (0.29 ; 3.14) 1	1.04 (0.62 ; 1.74) 1
**IgG3**	2656.49 (0.07;2006 695789.1) 0.79	0.63 (0.194;2.03) 1	1.72 (0.59;5.3) 1	1.18 (0.73;1.93) 1	1.27 (0.8;2.07) 1	1.13 (0.84;1.53) 1	1.02 (0.91;1.16) 1	1.22 (0.95;1.58) 0.69	2.09 (0.58;10.01) 1	0.42 (0.11;1.35) 0.82	0.7 (0.24 ; 1.98) 1	0.82 (0.46 ; 1.43) 1
**IgG4**	8.01 (0.01;1642 776.4) 1	0.86 (0.28;2.59) 1	1.76 (0.61;5.39) 1	1.08 (0.68;1.75) 1	1.24 (0.78;2.02) 1	1.08 (0.81;1.46) 1	1.05 (0.94;1.18) 1	1.18 (0.9;1.57) 0.84	1.97 (0.54;9.52) 1	0.43 (0.11;1.36) 0.82	0.87 (0.33 ; 2.36) 1	0.95 (0.56 ; 1.6) 1
**IgM**	0.74 (0.34;1.54) 1	1.98 (0.36;11) 1	1.72 (0.59;5.28) 1	0.99 (0.59;1.66) 1	1.24 (0.78;2) 1	1.04 (0.76;1.42) 1	1.11 (0.97;1.3) 0.71	1.17 (0.9;1.55) 0.84	1.82 (0.51;8.61) 1	0.42 (0.11;1.33) 0.82	0.94 (0.36 ; 2.5) 1	1.4 (0.64 ; 3.1) 1
